# Self-Assembled DNA Machine and Selective Complexation Recognition Enable Rapid Homogeneous Portable Quantification of Lung Cancer CTCs

**DOI:** 10.34133/research.0352

**Published:** 2024-04-18

**Authors:** Yue Wang, Congcong Shen, Chengyong Wu, Zixuan Zhan, Runlian Qu, Yi Xie, Piaopiao Chen

**Affiliations:** Department of Laboratory Medicine, Med+X Center for Manufacturing, Department of Respiratory and Critical Care Medicine, Targeted Tracer Research and Development Laboratory, National Clinical Research Center for Geriatrics, West China Hospital, Sichuan University, Chengdu, Sichuan 610041, China.

## Abstract

In this study, we systematically investigated the interactions between Cu^2+^ and various biomolecules, including double-stranded DNA, Y-shaped DNA nanospheres, the double strand of the hybridization chain reaction (HCR), the network structure of cross-linked HCR (cHCR), and small molecules (PPi and His), using Cu^2+^ as an illustrative example. Our research demonstrated that the coordination between Cu^2+^ and these biomolecules not only is suitable for modulating luminescent material signals through complexation reactions with Cu^2+^ but also enhances signal intensities in materials based on chemical reactions by increasing spatial site resistance and local concentration. Building upon these findings, we harnessed the potential for signal amplification in self-assembled DNA nanospheres and the selective complexation modulation of calcein in conjunction with the aptamer targeting mucin 1 as a recognition probe. We applied this approach to the analysis of circulating tumor cells, with the lung cancer cell line A549 serving as a representative model. Our assay, utilizing both a fluorometer and a handheld detector, achieved impressive detection limits of ag/ml and single-cell levels for mucin 1 and A549 cells, and this approach was successfully validated using 46 clinical samples, yielding 100% specificity and 86.5% sensitivity. Consequently, our strategy has paved the way for more portable and precise disease diagnosis.

## Introduction

Metal ions are prevalent in the biological world, playing a crucial role in applications ranging from bioanalysis to medical treatments. It is now understood that metal cations such as Cu^2+^, Ag^+^, Hg^2+^, Cd^2+^, Pd^2+^, and others exhibit strong coordination with functional groups like thiol, carboxylate, and amine groups of cysteine (Cys) [1,2], the imidazole group of histidine (His) [3,4], as well as the phosphate of pyrophosphate (PPi) [5]. These interactions have led to the development of a wide array of sensing platforms. Additionally, metal ions can interact with biomolecules, such as proteins and nucleic acids, holding important promise in the biomedical field [6]. Notably, DNA stands out due to its programmability, remarkable structural complexity, adjustability in sequence length, and ease of modification [7]. Over the years, DNA templates, with their specific coordination interactions with metal ions, have been used to synthesize sub-nanometer-sized DNA-templated metal nanoclusters such as Au NCs and Ag NCs [8,9]. Moreover, base pairs mediated by metal ions, especially T-Hg^2+^-T and C-Ag^+^-C, have been harnessed as recognition components [10,11]. Cytosine-rich I-motif structures and guanine-rich G-quadruplex structures have been utilized in disease detection and cancer treatment [12]. However, metal nanocluster synthesis can be challenging [13], Ag^+^ requires protection from light, and introducing Hg^2+^ could potentially pose an environmental pollution risk. Additionally, the stability and configuration of G-quadruplex structures are intricately linked to the presence of K^+^ and Na^+^ in the solution, thereby imposing constraints on their use in bioanalysis [14,15]. DNA-templated nanomaterials containing copper metal ions offer advantages due to their affordability, non-toxicity, and abundance of copper elements, although they face issues of poor photostability and weak emission intensities [16,17]. This low fluorescence stability is caused by the oxidation of copper due to reactions with dissolved oxygen and by radicals generated during the oxidation process [18]. Interestingly, previous research has shown that the introduction of high concentrations of histone and fructose can enhance fluorescence stability [19,20]. However, limited research has explored the interplay between Cu^2+^ and DNA and how this knowledge can be harnessed for bioanalysis, thus neglecting the issue of stability.

Liquid biopsy of tumors presents an appealing alternative to invasive tissue biopsy, offering direct and efficient detection through non-invasive sampling, ultimately facilitating early diagnosis and dynamic monitoring [21]. Circulating tumor cells (CTCs) have emerged as a promising biomarker for liquid tumor biopsies, as they carry valuable molecular and biological information regarding primary tumors and metastases [22–24]. Given the rarity of CTCs in peripheral blood, it becomes crucial to develop straightforward and efficient preprocessing methods along with highly sensitive quantification techniques. Several methods exist for CTCs separation, including immunomagnetic beads, ultrafine membrane filtration, and density gradient centrifugation, as well as innovative approaches like microfluidics [25–28]. In contrast to these complex methods, our team has developed a cost-effective, swift method for CTCs extraction [29]. This approach demands just 1 centrifugal machine, 2 reagents, and a 3-step centrifugation process, obtaining CTCs in under 45 min. To achieve trace-level cellular detection, a variety of nucleic acid and nanomaterial amplification strategies were employed, coupled with advanced instrumentation [30–32]. However, many of these methods still entail labor-intensive and expensive pre-treatment steps, further complicated by non-uniform assays, rendering convenient CTCs analysis a challenging endeavor. The T-Hg^2+^-T, C-Ag^+^-C hairpin structure-triggered cascade enzyme-free amplification has enabled homogeneous tumor diagnosis [11,33]. However, it is essential to address the light sensitivity of Ag^+^ and the health hazards associated with Hg^2+^. In recent years, advancements in DNA nanosphere structures have opened up new possibilities. These structures are obtained by predictable and programmable self-assembly of DNA molecules, which are multivalent and anisotropic, allowing the attachment of multiple signaling molecules and facilitating rapid signal amplification [34,35]. Besides, there have been no reports of cytotoxicity of DNA nanospheres on living cells. It should be noted that the nucleic acid sequences need to be annealed; therefore, the operation is not performed at room temperature. Leveraging the forces between Cu^2+^ and DNA, combined with enzyme-free nanospheres and aptamers, could potentially pave the way for an entirely new, simple, and environmentally friendly approach to CTCs analysis.

Herein, we examined the interactions between Cu^2+^ and DNA by exploiting the coordination of calcein and Cu^2+^, as well as the chemical reactions between cadmium telluride quantum dots (CdTe QDs) and Cu^2+^. We separately employed double-stranded DNA (dsDNA), DNA nanospheres, and biomolecules (PPi and His) to form complexes with Cu^2+^, demonstrating their modulation of calcein. Similar observations were made with QDs, with the exception that QDs remained unaffected by the Cu^2+^ and DNA complexes. To expand our applications, we integrated streptavidin (SA) with hybridization chain reaction (HCR) amplification to create cross-linked HCR (cHCR), which led to the regulation of QDs’ fluorescence signals by enhancing spatial site resistance and local concentration. With these discoveries, we targeted mucin 1 as a marker for homogeneous and portable CTCs detection in lung cancer blood samples within a 3-h timeframe. We achieved this by utilizing self-assembling DNA machines and selective complexation modulation of calcein. Our approach enabled detection limits for both mucin 1 and A549 cells at the ag/ml level and single-cell level, as verified by both a fluorometer and a handheld detector developed in-house. Simultaneously, we ensured the accurate detection of 46 clinical blood samples. As a result, we present a novel approach for utilizing DNA nanomaterials that simplifies their application in bioanalysis.

## Results

### Quantitative principles of CTCs

**Fig. 1.**
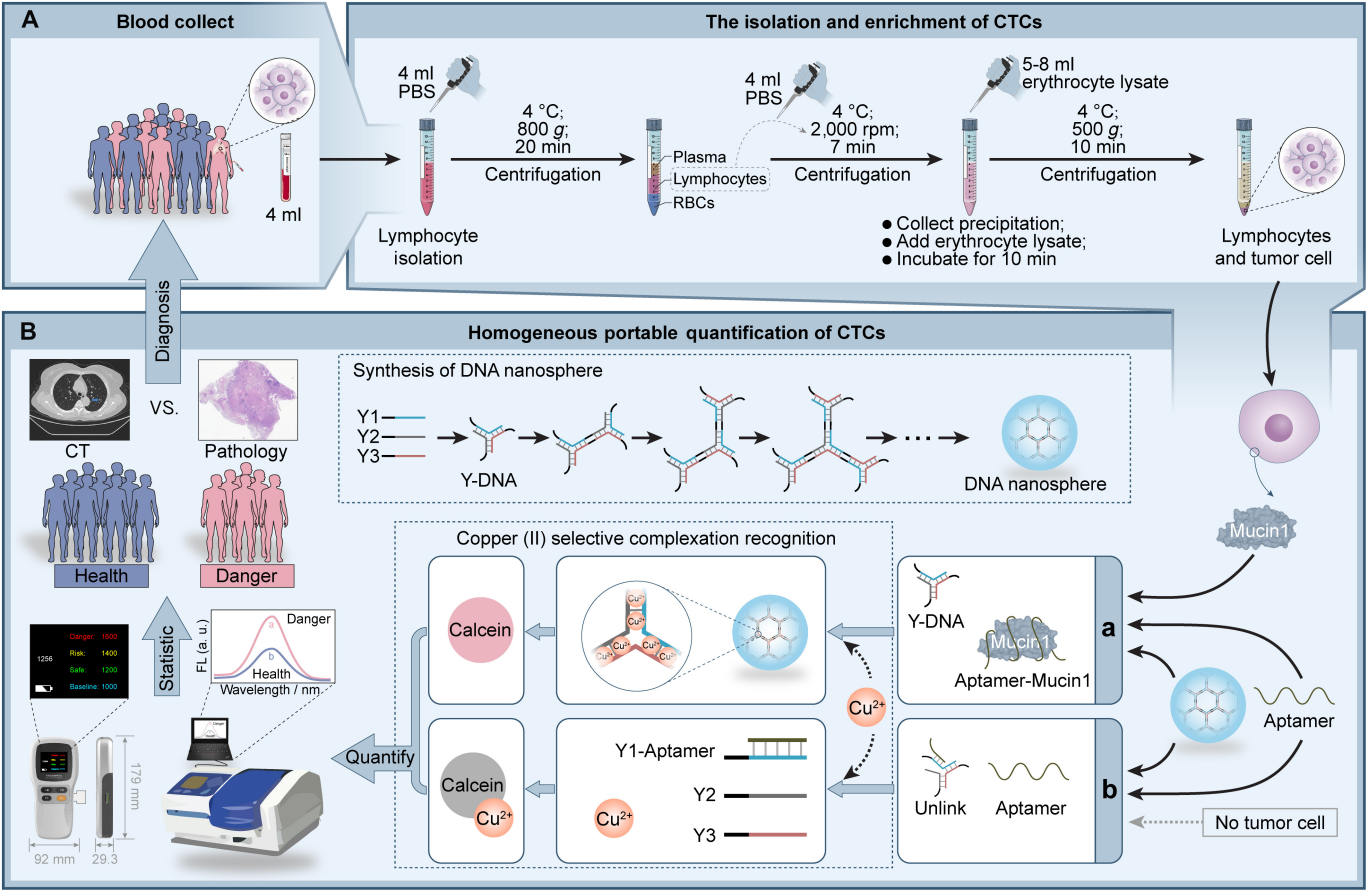
Schematic diagram of extraction and detection for CTCs. (A) Isolation and enrichment of CTCs from whole blood samples. (B) Rapid homogeneous detection of CTCs based on self-assembled DNA machine and selective complexation recognition.

The analysis of CTCs involved 2 primary components: efficient separation and homogeneous detection. Given the extremely low concentrations of CTCs in human blood, the primary emphasis was on optimizing CTCs separation. As depicted in Fig. 1A, a 3-step centrifugation method employing 2 separation solutions was employed. Initially, an equal volume of phosphate buffer solution (PBS) was added to the blood to reduce blood viscosity. Lymphocyte separation solution was introduced, followed by centrifugation. The second layer containing lymphocytes and the third layer with separation fluid were collected, mixed with an equal volume of PBS, and subjected to another round of centrifugation. The resulting precipitate was retained, and erythrocyte lysis solution was added to lyse any remaining erythrocytes. After the third centrifugation, the sediments in the tube represented the desired CTCs. To preserve the cells, 10% fetal bovine serum was applied.

Homogeneous portable quantification of CTCs involved the utilization of mucin 1, a highly expressed marker on the tumor cell surface, along with its aptamer probe (*K*_d_ = 0.135 nM) [36], self-assembled DNA machinery, selective Cu^2+^ complexation recognition, and handheld instrumentation for detection (Fig. 1B). To initiate this process, single-stranded DNA (ssDNA) Y1, Y2, and Y3, each bearing palindromic sequences (depicted as black lines), self-assembled to form DNA nanospheres through base complementary pairing. Notably, the palindromic sequence of Y1 bonded with itself, while Y2 and Y3 bonded with themselves and with each other. The longer palindromic sequence in Y1, compared to Y2 and Y3, provided more space for the subsequent binding of the aptamer to Y1 [37]. Cu^2+^ coordinated with the N7 and N1 atoms of purines (A and G), the O atom of the ring, the N3 atoms of pyrimidines (T and C), and the phosphorus oxygen atom of DNA to form inner-sphere complexes [38,39]. In the absence of the target mucin 1 or CTCs, the aptamer paired with Y1, disrupting the conformation of the DNA nanospheres and releasing Y2 and Y3. This resulted in an increase in free Cu^2+^, which, in turn, quenched the fluorescent signal of calcein. When mucin 1 or CTCs were present, they were specifically recognized and complexed with the aptamer, thereby retaining most of the DNA nanospheres. Cu^2+^ bound with the DNA nanosphere to create a DNA nanosphere–Cu^2+^ complex. In this context, the fluorescence of calcein remained unquenched because the interaction between Cu^2+^ and DNA was comparable to that of calcein, demonstrating selective complex recognition of Cu^2+^. Consequently, based on the marked difference in fluorescence signals between free Cu^2+^ and the DNA nanosphere–Cu^2+^ complex, high-sensitivity homogeneous analysis of mucin 1 and CTCs was achieved.

In terms of signal output methods, we employed both a fluorometer and a self-developed portable handheld fluorescence spectrometer, both of which yielded quantitative results. Notably, the handheld instrument enabled the simple collection of digital fluorescence intensity values for the analysis of mucin 1 or CTCs by transferring the reaction solution to the circular area of the test strip (Scheme S1) and subsequently measuring fluorescence at 510 nm. This approach was straightforward and efficient, enabling the rapid portable quantification of CTCs within a span of 3 h. Furthermore, given the non-dependent nature of Cu^2+^ binding with DNA, this method could find broader applications in disease diagnosis by altering the DNA self-assembly machinery’s sequence design.

### Cu^2+^ and DNA coordination for modulating signals of luminescent materials

**Fig. 2.**
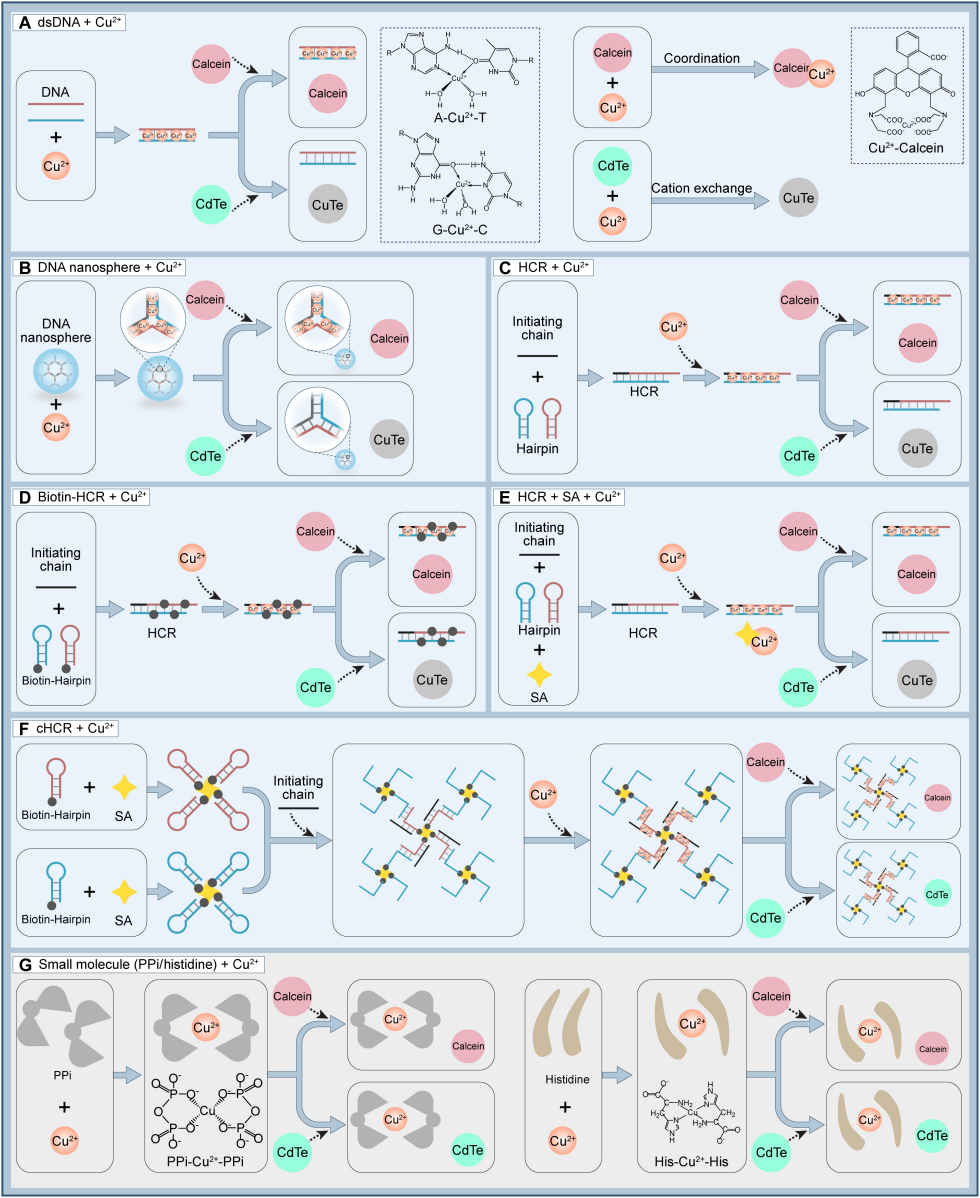
Schematic diagram of Cu^2+^ and DNA or small-molecule coordination applied to signal regulation of luminescent materials. (A) dsDNA + Cu^2+^. (B) DNA nanosphere + Cu^2+^. (C) HCR + Cu^2+^. (D) Biotin-HCR + Cu^2+^. (E) HCR + SA + Cu^2+^. (F) cHCR + Cu^2+^. (G) Small molecule (PPi/histidine) + Cu^2+^.

**Fig. 3.**
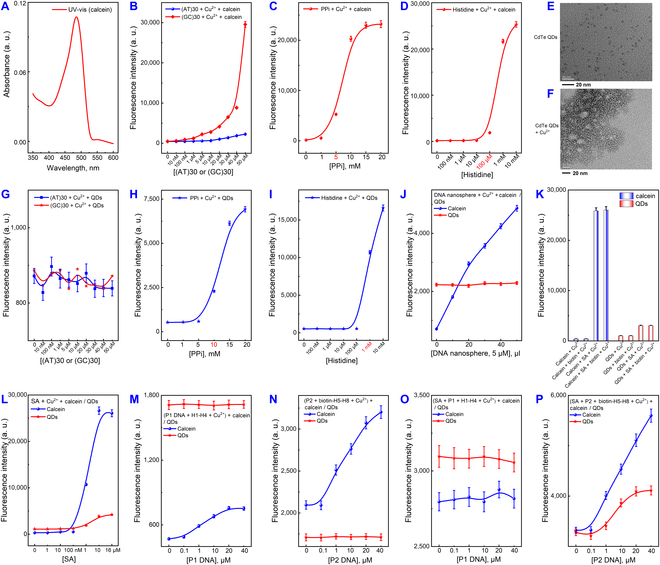
Cu^2+^ and DNA coordination for modulating signals of luminescent materials. (A) UV–vis absorption of calcein. The influence of (B) dsDNA (AT/GC) + Cu^2+^, (C) PPi + Cu^2+^, and (D) His + Cu^2+^ on the fluorescence of calcein. TEM images of (E) CdTe QDs, and (F) CdTe QDs + Cu^2+^. The influence of (G) dsDNA (AT/GC) + Cu^2+^, (H) PPi + Cu^2+^, and (I) His + Cu^2+^ on the fluorescence of CdTe QDs. (J) The influence of DNA nanospheres + Cu^2+^ on the fluorescence of calcein and CdTe QDs. (K) The influence of SA/biotin + Cu^2+^ on the fluorescence signals of calcein and CdTe QDs. (L) The influence of different concentrations of SA reacting with Cu^2+^ on the fluorescence of calcein and CdTe QDs. The influence of (M) HCR + Cu^2+^, (N) biotin-HCR + Cu^2+^, (O) SA + HCR + Cu^2+^, and (P) cHCR + Cu^2+^ on the fluorescence signals of calcein and CdTe QDs. The error bars were derived from 3 repeated measurements.

As illustrated in Figs. 2 and 3, we explored the modulation of luminescent materials (calcein and CdTe QDs) through the coordination of Cu^2+^ with DNA and biomolecules (PPi and His). Calcein, having similar coordination with Cu^2+^, and QDs, which undergo a chemical reaction, were the focal points of our investigation (Fig. 2A). The characteristic UV absorption peaks of calcein were observed at 480 nm (Fig. 3A). In the presence of Cu^2+^, the signal of calcein increased as the concentration of dsDNA rose (Fig. 3B). Similar trends were noted for PPi and His (Fig. 3C and D). This indicated that after Cu^2+^ complexed with dsDNA and PPi/His, the remaining Cu^2+^ quenched the fluorescence of calcein, while the Cu^2+^ used for coordination hardly participated (Fig. 2G). Consequently, calcein modulation could be accomplished by adjusting the quantity of the coordination complex. Interestingly, dsDNA-Cu^2+^ exhibited better sensitivity in regulating calcein compared to PPi (5 mM) and His (100 μM). Afterwards, we transitioned to using QDs as signaling molecules and characterized it (Fig. 3E). QDs exhibited agglomeration due to the cation exchange reaction (CER) with Cu^2+^ (Fig. 3F). Notably, dsDNA-Cu^2+^ was not effective in modulating QDs (Fig. 3G), but PPi-Cu^2+^-PPi and His-Cu^2+^-His showed effectiveness (Fig. 3H and I). We then replaced dsDNA with DNA nanospheres, which could accommodate larger amounts of Cu^2+^ (Fig. 2B). Here, even a small amount of nanospheres had a substantial impact on the calcein signal, while QDs remained unresponsive (Fig. 3J). This phenomenon suggested that the interaction between Cu^2+^ and DNA was weaker than the ionic bond and the coordination force between Cu^2+^ and small molecules. We then attempted to create 4-armed DNA nanostructures using SA and 4 biotin hairpin DNA probes to form a hydrogel network with high spatial site resistance for signaling modulation through cHCR (Fig. 2F). Initially, we tested the interference of Cu^2+^ from SA and biotin. As depicted in Fig. 3K and L, both fluorescence signal values increased after the addition of SA, indicating SA’s substantial adsorption of Cu^2+^, while biotin had minimal interference. We carefully adjusted the amounts of SA and Cu^2+^ to eliminate interference and compared normal HCR with cHCR. As anticipated, only the successful execution of cHCR, forming a reticulated polymer, could modulate calcein and QDs (Fig. 3P). Neither normal HCR nor the addition of SA or biotin in isolation affected QDs (Figs. 3M to O and 2C to E). Based on these experimental findings, we concluded that the coordination between DNA and Cu^2+^ could be employed to modulate luminescent materials through coordination. It could also be applied to modulate luminescent materials through chemical reactions by increasing spatial site resistance and local concentration. This opens up new possibilities for applying Cu^2+^ with DNA nanomaterials.

**Fig. 4.**
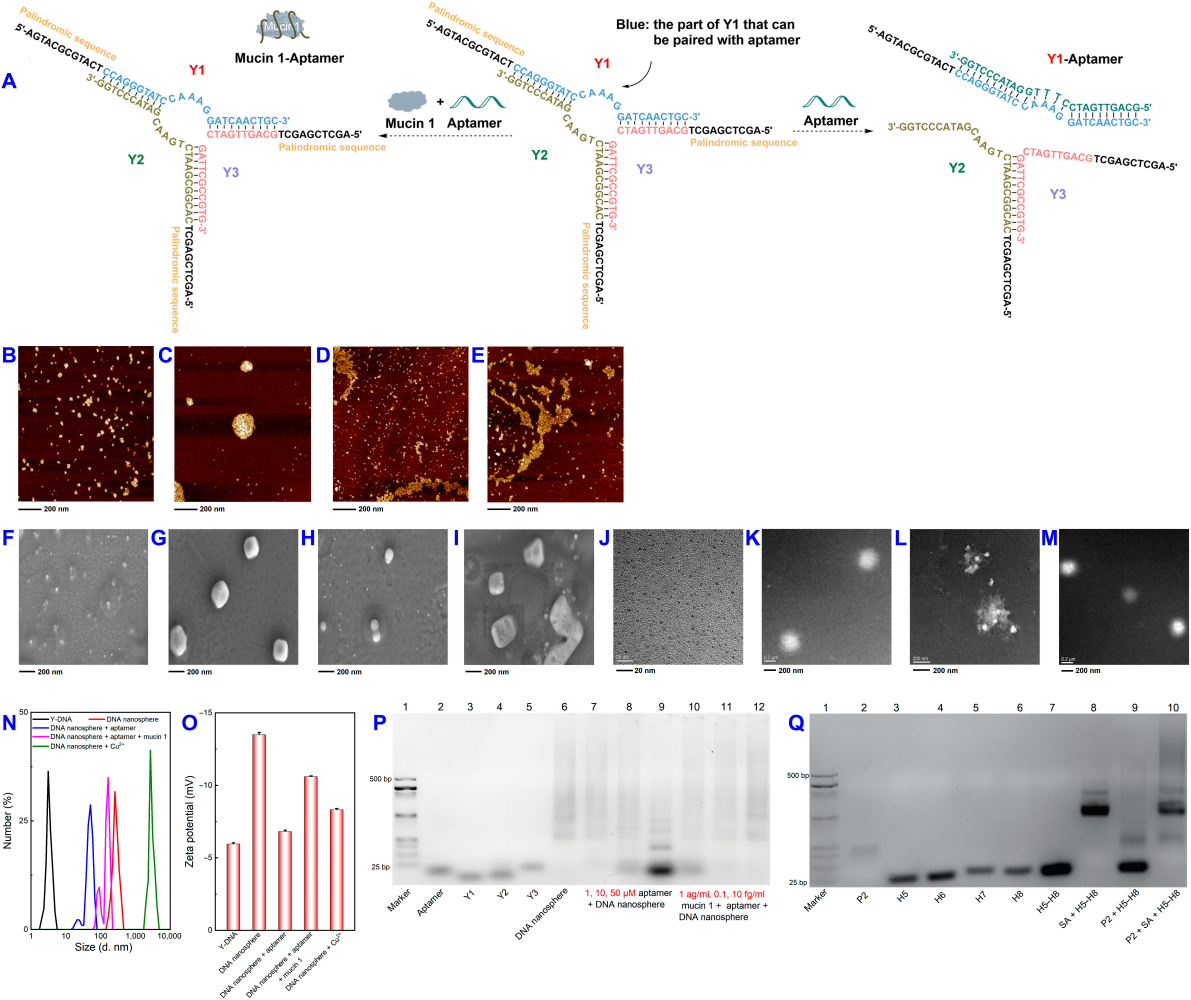
Feasibility of DNA nanospheres for CTCs analysis. (A) Assembly process of Y-DNA. AFM (B to E), SEM (F to I), and TEM (J to M) images of Y-DNA, DNA nanosphere, DNA nanosphere + aptamer, and DNA nanosphere + aptamer + mucin 1, respectively. DLS (N) and Zeta potential (O) of Y-DNA, DNA nanosphere, DNA nanosphere + aptamer, DNA nanosphere + aptamer + mucin 1, and DNA nanosphere + Cu^2+^. Agarose gel electrophoresis of DNA nanospheres (P) and cHCR (Q) under different conditions. The error bars were derived from 3 repeated measurements.

Based on the modulation achieved with DNA nanospheres combined with Cu^2+^ for calcein, we harnessed this capability for the analysis of CTCs, leveraging the simplicity and self-assembly properties of DNA nanospheres. The DNA nanomachines, comprising Y-monomers as components, involved 3 ssDNA sequences (Y1, Y2, and Y3), each possessing palindromic sequences (represented by black sections) that hybridized with one another to form Y-DNAs (Fig. 4A). This, in turn, led to the formation of DNA nanospheres. To investigate the size and morphology of the DNA nanospheres under different conditions, we employed atomic force microscopy (AFM), scanning electron microscopy (SEM), and transmission electron microscopy (TEM). As depicted in Fig. 4B to E, the AFM images revealed that Y-DNA monomers without palindromic sequences were small and uniformly distributed. In contrast, the DNA nanospheres exhibited a clear spherical structure. Upon the addition of the aptamer, the structure of the DNA nanospheres was disrupted. With further introduction of mucin 1, the aptamer interacted with the protein, resulting in the retention of the spherical structure. Similar observations were made for SEM (Fig. 4F to I) and TEM (Fig. 4J to M). To corroborate these findings, dynamic light scattering (DLS) and zeta potential measurements were conducted (Fig. 4N and O). These measurements indicated that the average diameters of Y-DNA, DNA nanospheres, DNA nanospheres + aptamer, and DNA nanospheres + aptamer + mucin 1 were 3.20 nm, 273.10 nm, 48.37 nm, and 145.10 nm, respectively. Conversely, the introduction of Cu^2+^ into the DNA nanospheres led to a increase in diameter to 2,885.42 nm, accompanied by an elevation in zeta potential, indicating the formation of Cu^2+^–DNA nanosphere complexes. Additionally, experiments involving agarose gel electrophoresis demonstrated the reaction process of the aptamer and mucin 1 with DNA nanospheres (Fig. 4P), validating the system’s suitability for mucin 1 analysis. Figure 4Q illustrated the electrophoretic outcomes of cHCR, which successfully generated high-molecular-weight products when SA and initiating chain P2 were introduced (lane 10), thus allowing the modulation of nanomaterials exhibiting stronger force interaction with Cu^2+^.

### Analytical performance of mucin 1

After optimizing the experimental conditions (Fig. S2), we evaluated the performance of the mucin 1 analysis system. Within the concentration range of 10 ag/ml to 10 fg/ml, there was a strong linear relationship between the fluorescence signal of calcein and the logarithm of the concentration (Fig. S3A to C). The limit of detection (LOD) was determined to be 3 ag/ml at a signal-to-noise ratio of 3:1 [40]. In comparison to previously published research (Table S2), our assay demonstrated a lower detection limit primarily attributed to the precise design of the DNA sequences for self-assembling nanospheres and the binding of DNA nanosphere structures to Cu^2+^. Furthermore, the enzyme-free, homogeneous, and straightforward nature of the procedure allowed its completion within 3 h.

To facilitate fast and accurate measurements, we developed an independently designed handheld fluorometer (Fig. S3D). It operated on the same principles as a conventional fluorometer but utilized a light-emitting diode as the light source. Measurement of calcein fluorescence was accomplished by adjusting the light source, and the fluorescence value was rapidly read at a specific point within the emission wavelength range, such as 510 nm. Considering the calcein loading capacity and storage difficulty of the paper, Whatman chromatography paper with no background fluorescence was selected for testing. The completed reaction was detected by placing the calcein-containing solution in the corresponding designated circular area on the test strip, demonstrating good linearity over the mucin 1 concentration range of 10 ag/ml to 10 fg/ml (Fig. S3E and F), with an LOD of 4 ag/ml. Portable testing was achieved without sacrificing sensitivity when compared to larger fluorometers.

The specificity of our approach was assessed by the detection of non-target proteins. The results indicated that even at higher concentrations (10 fg/ml), potentially interfering proteins had a minimal effect (in comparison to the blank) in the detection system, including homologous proteins to mucin 1 (Fig. S3G). This finding provided the foothold for the subsequent detection of clinical CTCs.

### Analytical performance of A549 cells

**Fig. 5.**
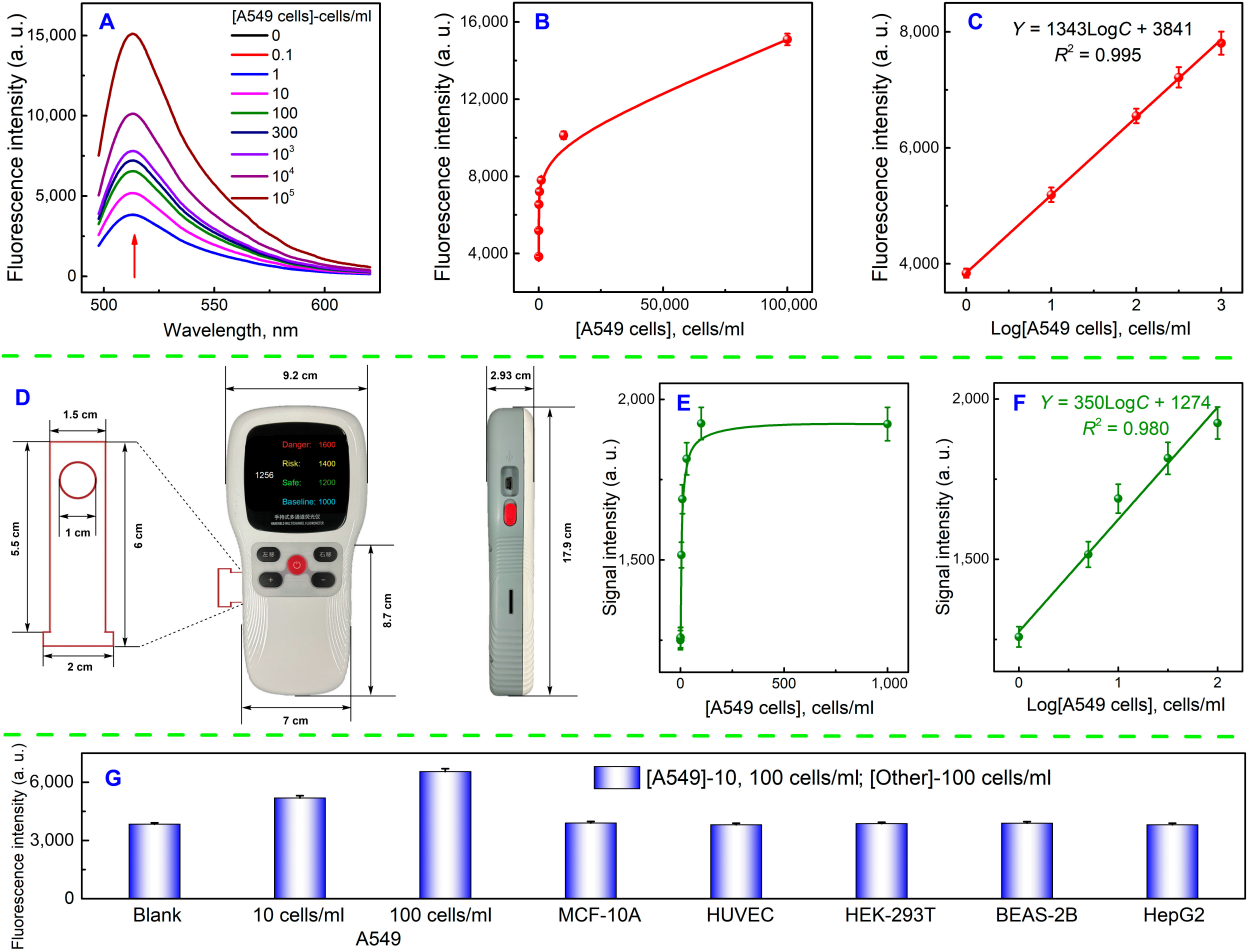
Analytical performance and specificity of A549 cells. (A) Fluorescence spectrum of calcein with different concentrations of A549 cells. (B and C) Fluorescence values and linear fitting of A549 cells by fluorometer. (D) Dimensions of handheld fluorometer and test strips. (E and F) Fluorescence values and linear fitting of A549 cells by a handheld fluorometer. (G) Selectivity analysis of various types of cells. The error bars were calculated from 3 repetitions of the experiment.

Building upon promising previous experimental results, we extended our strategy to the cellular level. Given the overexpression of mucin 1 in lung cancer CTCs, we utilized A549 cells as a model to measure mucin 1 on their surface, thus indirectly assessing the cellular concentration. The fluorescence signals exhibited a strong linear relationship with the logarithm of the concentration within the range of 1 to 10^3^ cells/ml (Fig. 5A to C), with the LOD calculated to be 1 cell/ml based on a triple signal-to-noise ratio. Achieving detection at the single-cell level was easily realized, surpassing the capabilities of previous CTCs assays (Table S3). Similarly, standard curves for cell concentration were established using a handheld fluorometer, enabling swift readings of fluorescence values following the completion of the reaction. The linear range spanned 1 to 100 cells/ml, with an LOD of 2 cells/ml, demonstrating comparable performance to that of the large fluorometer. Moreover, an approximate quantification of cell concentration was feasible using the linear equation (Fig. 5E and F). To evaluate the specificity of the method, we examined various types of cells that are not expressing mucin 1 (Fig. 5G). The signal of negative cells at 100 cells/ml was comparable to that of the blank solution. Conversely, the presence of A549 cells elevated the fluorescence values, substantiating the method’s high specificity.

Summarizing the above analytical performance, our strategy entailed a straightforward enrichment step and an accurate, sensitive dual signal output mode. The 3-step centrifugation pre-processing step ensured thorough and gentle enrichment of CTCs. Additionally, the economic production and long-term preservation of testing strips, obtained by printing and cutting from chromatography paper, added to the simplicity of the operation. Furthermore, the development of a self-designed handheld fluorometer facilitated rapid field inspection. Thus, our strategy holds promise for the examination of clinical samples for CTCs.

### Clinical application of the method

**Fig. 6.**
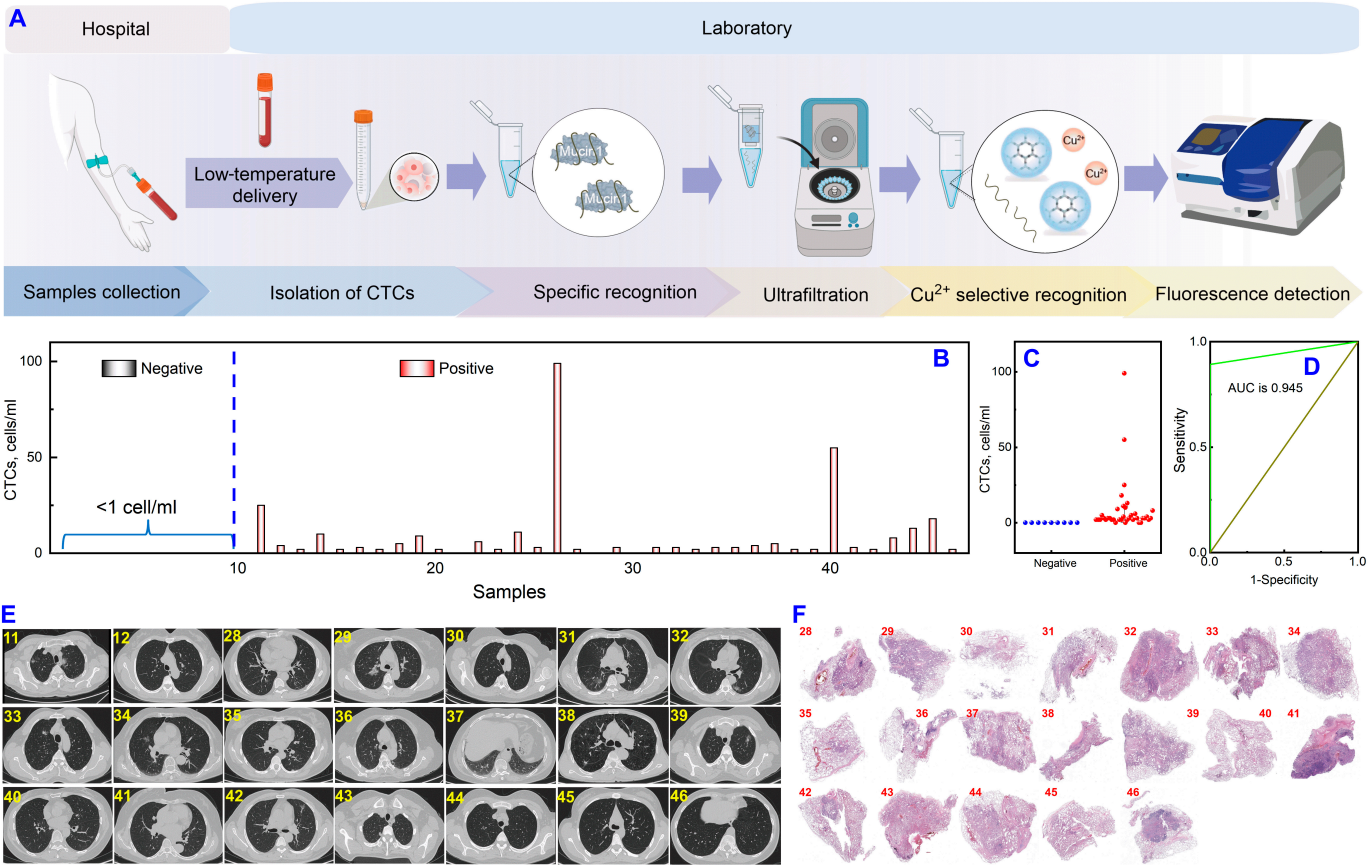
Flow chart and analysis results of blood samples from lung cancer patients by a fluorometer. (A) Workflow diagram for clinical blood sample analysis. (B) Analysis results of CTCs. Scatter plot (C) and ROC analysis (D) of CTCs concentration. Clinical CT (E) and pathological biopsy results (F). Error lines were derived from 3 repeated tests.

We assessed the clinical feasibility and accuracy of our strategy by applying it to 46 clinical whole blood samples from lung cancer patients (*n* = 37) and healthy volunteers (*n* = 9) for the detection of CTCs using fluorometry. The process of analyzing clinical samples was depicted in Fig. 6A. Initially, CTCs were isolated from whole blood according to the sample pre-treatment procedure described earlier (Fig. 1A). Subsequently, the CTCs were co-incubated with the aptamer to facilitate ample binding. The solution was centrifuged in a 50-kDa ultrafiltration tube to remove interference from free mucin 1, red and white blood cells. The solution in the outer tube was collected to obtain the remaining unbound aptamer. It was then sequentially subjected to reaction with DNA nanospheres and Cu^2+^, and finally, the assay was completed using the fluorometer. As presented in Fig. 6B and Table S4, the concentration of CTCs in the negative samples (No. 1-9) was below 1 cell/ml (outside the linear range of the method), whereas the concentration of CTCs in the positive patient samples (No. 10-46) exceeded 1 cell/ml, with the exception of 5 samples. The specificity and sensitivity were determined to be 100% (9/9) and 86.5% (32/37), respectively. A scatter plot (Fig. 6C) and receiver operating characteristic (ROC) analysis (Fig. 6D) revealed a difference between healthy volunteers and patients, with an area under the curve of 0.945, indicating the method’s strong clinical diagnostic value. Furthermore, a high level of concordance between the analysis results of CTCs and the clinical diagnosis was observed (Fig. 6E and F), validating the clinical feasibility and reliability of the strategy we developed.

**Fig. 7.**
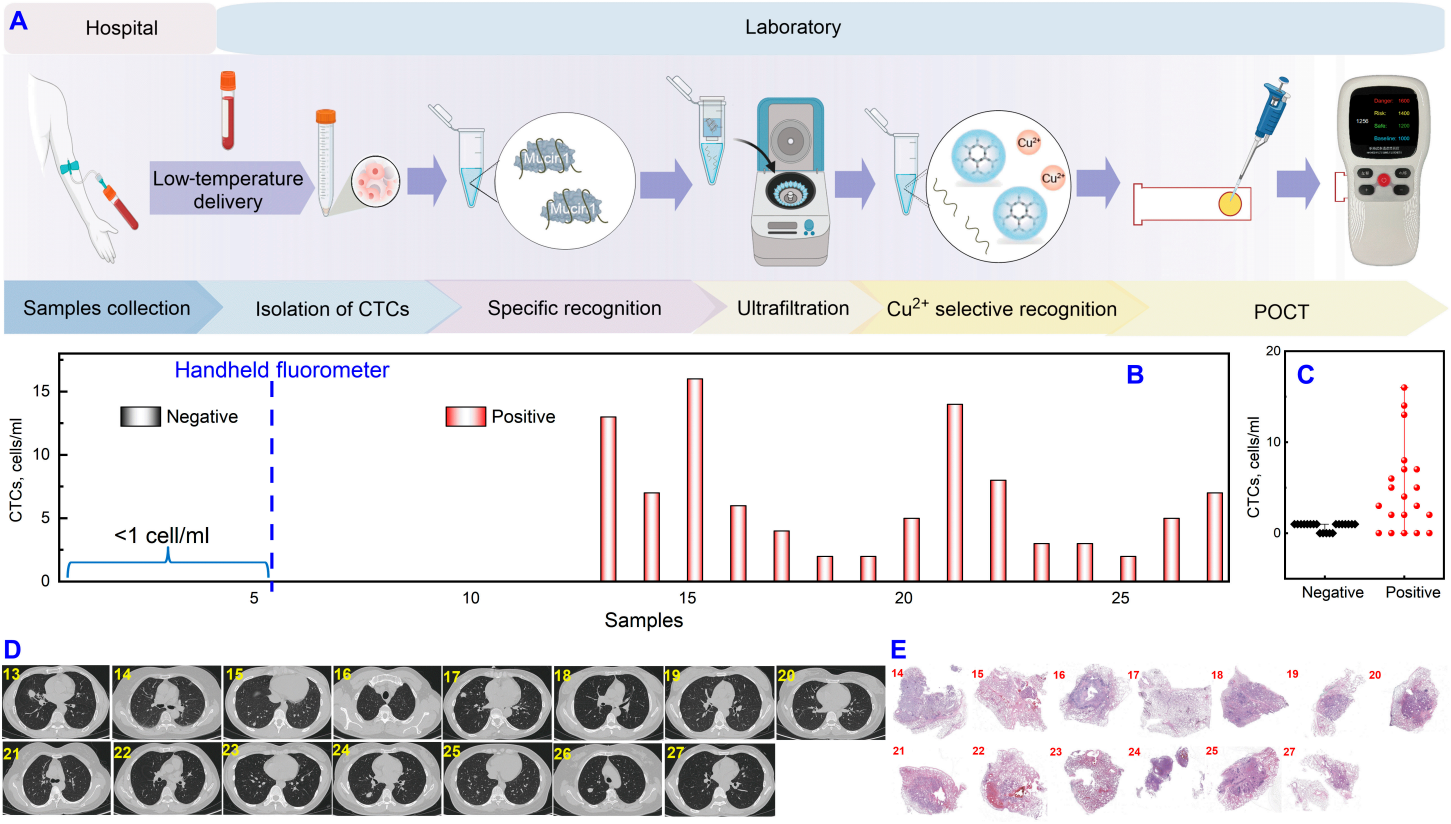
Flow chart and analysis results of blood samples from lung cancer patients by a handheld fluorometer. (A) Workflow diagram for field inspection of clinical blood samples. (B) CTCs results obtained by a handheld fluorometer. (C) Scatter plot of CTCs concentration. Clinical CT (D) and pathological biopsy results (E). Error lines were derived from 3 repeated tests.

While fluorometers ensure accurate quantification, their bulky size, high cost, and demanding maintenance render them unsuitable for household or field inspection. In comparison, the independently developed handheld fluorometer offered remarkable advantages. Using the same sample processing and measurement method as previously described, a mere drop of the reaction solution was placed in the corresponding circle on the test paper for immediate readout (Fig. 7A). The handheld fluorometer demonstrated strong agreement with the clinical diagnosis for the 5 negative samples and 15 positive samples (Fig. 7B, No. 1-5 and No. 13-27 of Table S4). Notably, there was a difference between healthy volunteers and patients (Fig. 7C). Clinical CT and pathological biopsy result further underscored the reliability of our findings (Fig. 7D and E). In addition, the reproducibility and stability of the instrument were demonstrated through multiple tests and multiple time tests (Fig. S4). The clinical folate receptor-polymerase chain reaction (FR-PCR) kit (clinical critical value 8.7 FU/3 ml) was further compared with the fluorescence results, and the good consistency (Table S5) displayed the applicability of this method. These results indicate that the proposed strategy enables prompt, sensitive, and accurate diagnosis of clinical lung cancer samples, offering a promising new portable and valuable modality for clinical examination.

## Discussion

In conclusion, leveraging the modulation of luminescent materials via the coordination force between Cu^2+^ and DNA, we successfully achieved homogeneous and portable detection of CTCs in lung cancer blood samples within 3 h using self-assembled DNA machines and selective complexation recognition of calcein. Our strategy was validated using both a fluorometer and a self-developed, portable handheld fluorescence spectrometer, both of which yielded quantitative results, with detection limits for both mucin 1 and A549 cells achieved at the ag/ml and single-cell levels, respectively. Our strategy was proven feasible and reliable in the analysis of 46 clinical samples from lung cancer patients, demonstrating excellent sensitivity and specificity. The results obtained from the clinical blood samples were consistent with CT imaging and pathological findings. Therefore, by focusing on the non-dependent binding of Cu^2+^ with DNA and the generalizability of selective coordination, our method holds promise for broader applications in disease diagnosis through the modification of the DNA self-assembly machine and signaling reporter materials.

## Materials and Methods

### Analysis steps of A549 cells

First, 10 μl of 12 μM aptamer-DNA, 1 ml of A549 cells at varying concentrations, and 40 μl of DNA nanospheres were combined in 70 μl of 10 mM 3-(*N*-morpholino) propanesulfonic acid buffer (containing 100 mM NaNO_3_, pH 7.6). This mixture was then subjected to agitation in a shaker for 60 min. Following this, 10 μl of 5 μM CuSO_4_ solution was introduced to the reaction solution, and further agitation in the shaker for 50 min ensued. Finally, 6 μl of 10 μM calcein disodium salt was added, and the fluorescence value was measured 2 min after this final addition.

## Data Availability

Data are available from the corresponding author upon request.

## References

[1] Zhang X, Yan LG, Li J, Yu HQ. Adsorption of heavy metals by L-cysteine intercalated layered double hydroxide: Kinetic, isothermal and mechanistic studies. J Colloid Interface Sci. 2020;562:149–158. 31838351 10.1016/j.jcis.2019.12.028

[2] Laabd M, Imgharn A, Hsini A, Naciri Y, Mobarak M, Szunerits S, Boukherroub R, Albourine A. Efficient detoxification of Cr(VI)-containing effluents by sequential adsorption and reduction using a novel cysteine-doped PANi@Faujasite composite: Experimental study supported by advanced statistical physics prediction. J Hazard Mater. 2022;422: Article 126857. 34399223 10.1016/j.jhazmat.2021.126857

[3] Xian WJ, Hennefarth MR, Lee MW, Do T, Lee EY, Alexandrova AN, Wong GCL. Histidine-mediated ion specific effects enable salt tolerance of a pore-forming marine antimicrobial peptide. Angew Chem Int Ed. 2022;61(25): Article e202108501. 10.1002/anie.202108501 PMC9189074 35352449

[4] Parthasarathy S, Long F, Miller Y, Xiao YL, McElheny D, Thurber K, Ma B, Nussinov R, Ishii Y. Molecular-level examination of Cu^2+^ binding structure for amyloid fibrils of 40-residue Alzheimer’s beta by solid-state NMR spectroscopy. J Am Chem Soc. 2011;133(10):3390–3400. 21341665 10.1021/ja1072178 PMC3074258

[5] Rogers LB, Reynolds CA. Interaction of pyrophosphate ion with certain multivalent cations in aqueous solutions. J Am Chem Soc. 1949;71:2081–2085.

[6] Naskar S, Guha R, Mueller J. Metal-modified nucleic acids: Metal-mediated base pairs, triples, and tetrads. Angew Chem Int Ed. 2020;59(4):1397–1406. 10.1002/anie.201905913 31259475

[7] Gentile S, Del Grosso E, Prins LJ, Ricci F. Reorganization of self-assembled DNA-based polymers using orthogonally addressable building blocks. Angew Chem Int Ed. 2021;60(23):12911–12917. 10.1002/anie.202101378 33783934

[8] Xiao Y, Wu ZN, Yao QF, Xie JP. Luminescent metal nanoclusters: Biosensing strategies and bioimaging applications. Aggregate. 2021;2:114–132.

[9] Liu B, Qi ZL, Chao J. Framework nucleic acids directed assembly of Au nanostructures for biomedical applications. Interdiscip Med. 2023;1: Article e20220009.

[10] Porchetta A, Vallée-Bélisle A, Plaxco KW, Ricci F. Allosterically tunable, DNA-based switches triggered by heavy metals. J Am Chem Soc. 2013;135(36):13238–13241. 23971651 10.1021/ja404653q PMC3831027

[11] Chen PP, Wang Y, He YQ, Huang K, Wang X, Zhou RH, Liu TYH, Qu RL, Zhou J, Peng W, et al. Homogeneous visual and fluorescence detection of circulating tumor cells in clinical samples *via* selective recognition reaction and enzyme-free amplification. ACS Nano. 2021;15(7):11634–11643. 34129315 10.1021/acsnano.1c02080

[12] Ge J, Qi ZY, Zhang LL, Shen XP, Shen YM, Wang WX, Li ZH. Label-free and enzyme-free detection of MicroRNA based on a hybridization chain reaction with hemin/G-quadruplex enzymatic catalysis-induced MoS_2_ quantum dots *via* the inner filter effect. Nanoscale. 2020;12(2):808–814. 31830179 10.1039/c9nr08154b

[13] Kailasa SK, Borse S, Koduru JR, Murthy ZVP. Biomolecules as promising ligands in the synthesis of metal nanoclusters: Sensing, bioimaging and catalytic applications. Trends Envion Anal Chem. 2021;32: Article e00140.

[14] Chen JY, Hickey BL, Wang LL, Lee J, Gill AD, Favero A, Pinalli R, Dalcanale E, Hooley RJ, Zhong WW. Selective discrimination and classification of G-quadruplex structures with a host-guest sensing array. Nat Chem. 2021;13(5):488–495. 33795843 10.1038/s41557-021-00647-9

[15] Wang QY, Yang SF, Li FY, Ling DS. Nature-inspired K^+^-sensitive imaging probes for biomedical applications. Interdiscip Med. 2023;1: Article e20220004.

[16] Ouyang XY, Wang MF, Guo LJ, Cui CJ, Liu T, Ren YA, Zhao Y, Ge ZL, Guo XN, Xie G, et al. DNA nanoribbon-templated self-assembly of ultrasmall fluorescent copper nanoclusters with enhanced luminescence. Angew Chem Int Ed. 2020;59(39):11836–11844. 10.1002/anie.202003905 32267600

[17] Zhang L, Cai QY, Li J, Ge J, Wang JY, Dong ZZ, Li ZH. A label-free method for detecting biothiols based on poly(thymine)-templated copper nanoparticles. Biosens Bioelectron. 2015;69:77–82. 25703731 10.1016/j.bios.2015.02.012

[18] Rotaru A, Dutta S, Jentzsch E, Gothelf K, Mokhir A. Selective dsDNA-templated formation of copper nanoparticles in solution. Angew Chem Int Ed. 2010;49(1):5665–5667. 10.1002/anie.200907256 20629055

[19] Kim S, Lee ES, Cha BS, Park KS. High fructose concentration increases the fluorescence stability of DNA-templated copper nanoclusters by several thousand times. Nano Lett. 2022;15(15):6121–6127. 10.1021/acs.nanolett.2c01287 35895973

[20] Lian JY, Liu Q, Jin Y, Li BX. Histone-DNA interaction: An effective approach to improve the fluorescence intensity and stability of DNA-templated Cu nanoclusters. Chem Commun. 2017;53(93):12568–12571. 10.1039/c7cc07424g 29119169

[21] Bao H, Min L, Bu FQ, Wang ST, Meng JX. Recent advances of liquid biopsy: Interdisciplinary strategies toward clinical decision-making. Interdiscip Med. 2023;1: Article e20230021.

[22] Ring A, Nguyen-Straeuli BD, Wicki A, Aceto N. Biology, vulnerabilities and clinical applications of circulating tumour cells. Nat Rev Cancer. 2023;23(2):95–111. 36494603 10.1038/s41568-022-00536-4 PMC9734934

[23] Zhang C, Li ZD, Liu J, Liu C, Zhang HQ, Lee WG, Yao CY, Guo H, Xu F. Synthetic gene circuit-based assay with multilevel switch enables background-free and absolute quantification of circulating tumor DNA. Research. 2023;6:0217. 37789988 10.34133/research.0217 PMC10543738

[24] Bathini S, Raju D, Badilescu S, Kumar A, Ouellette RJ, Ghosh A, Packirisamy M. Nano-bio interactions of extracellular vesicles with gold nanoislands for early cancer diagnosis. Research. 2018;2018:3917986–3917986. 31549028 10.1155/2018/3917986 PMC6750071

[25] Wu LL, Ding HM, Qu X, Shi XN, Yang JM, Huang MJ, Zhang JL, Zhang HM, Song J, Zhu L, et al. Fluidic multivalent membrane nanointerface enables synergetic enrichment of circulating tumor cells with high efficiency and viability. J Am Chem Soc. 2020;142(10):4800–4806. 32049531 10.1021/jacs.9b13782

[26] Gao QQ, Zhao YM, Xu KL, Zhang C, Ma Q, Qi LQ, Chao DD, Zheng TT, Yang LL, Miao YY, et al. Highly specific, single-step cancer cell isolation with multi-aptamer-mediated proximity ligation on live cell membranes. Angew Chem Int Ed. 2020;59(52):23564–23568. 10.1002/anie.202011198 32896066

[27] Li M, Ding HM, Lin MH, Yin FF, Song L, Mao XH, Li F, Ge ZL, Wang LH, Zuo XL, et al. DNA framework-programmed cell capture *via* topology-engineered receptor-ligand interactions. J Am Chem Soc. 2019;141(47):18910–18915. 31691568 10.1021/jacs.9b11015

[28] Li Z, Wang GL, Shen Y, Guo NN, Ma N. DNA-templated magnetic nanoparticle-quantum dot polymers for ultrasensitive capture and detection of circulating tumor cells. Adv Funct Mater. 2018;28:1707152.

[29] Wang Y, Chen X, Shen X, He YQ, Zhan ZX, Liu CX, Xie Y, Lin F, Huang K, Chen PP. Simplified rapid enrichment of CTCs and selective recognition prereduction enable a homogeneous ICP-MS liquid biopsy strategy of lung cancer. Anal Chem. 2023;95(38):14244–14252. 37705297 10.1021/acs.analchem.3c02302

[30] Chen J, Tang JL, Meng HM, Liu Z, Wang L, Geng X, Wu YN, Qu LB, Li ZH. Recognition triggered assembly of split aptamers to initiate a hybridization chain reaction for wash-free and amplified detection of exosomes. Chem Commun. 2020;56(63):9024–9027. 10.1039/d0cc02337j 32639506

[31] Pan D, Fang ZZ, Yang EL, Ning ZQ, Zhou Q, Chen KY, Zheng YJ, Zhang YJ, Shen YF. Facile preparation of WO_3-x_ dots with remarkably low toxicity and uncompromised activity as co-reactants for clinical diagnosis by electrochemiluminescence. Angew Chem Int Ed. 2020;59(38):16747–16754. 10.1002/anie.202007451 32524717

[32] Song YL, Shi YZ, Huang MJ, Wang W, Wang Y, Cheng J, Lei ZC, Zhu Z, Yang CY. Bioinspired engineering of a multivalent aptamer-functionalized nanointerface to enhance the capture and release of circulating tumor cells. Angew Chem Int Ed. 2019;58(8):2236–2240. 10.1002/anie.201809337 30548959

[33] Jiang PJ, Bai YJ, Yan L, Feng P, Huang K, Chen J, Chen PP. Nanoarchitectonics-assisted simultaneous fluorescence detection of urinary dual miRNAs for noninvasive diagnosis of prostate cancer. Anal Chem. 2023;95(19):7676–7684. 37129316 10.1021/acs.analchem.3c00701

[34] Xu Z, Shi TH, Mo FY, Yu WQ, Shen Y, Jiang QY, Wang FA, Liu XQ. Programmable assembly of multivalent DNA-protein superstructures for tumor imaging and targeted therapy. Angew Chem Int Ed. 2022;61(44): Article e202211505. 10.1002/anie.202211505 36082964

[35] Li C-z, Hu TY. Hu TY, nanotechnology powered CRISPR-Cas systems for point of care diagnosis and therapeutic. Research. 2022;2022:9810237. 36157513 10.34133/2022/9810237 PMC9484831

[36] Ferreira CSM, Matthews CS, Missailidis S. DNA aptamers that bind to MUC1 tumour marker: Design and characterization of MUC1-binding single-stranded DNA aptamers. Tumour Biol. 2006;27(6):289–301. 17033199 10.1159/000096085

[37] Wang JL, Li J, Chen Y, Liu RT, Wu YX, Liu JB, Yang XH, Wang KM, Huang J. Size-controllable and self-assembled DNA nanosphere for amplified microRNA imaging through ATP-fueled cyclic dissociation. Nano Lett. 2022;22(20):8216–8223. 36194690 10.1021/acs.nanolett.2c02934

[38] Clever GH, Kaul C, Carell T. DNA-metal base pairs. Angew Chem Int Ed. 2007;46(33):6226–6236. 10.1002/anie.200701185 17640011

[39] Burda JV, Šponer J, Leszczynski J, Hobza P. Interaction of DNA base pairs with various metal cations (Mg^2+^, Ca^2+^, Sr^2+^, Ba^2+^, Cu^+^, Ag^+^, Au^+^, Zn^2+^, Cd^2+^, and Hg^2+^): Nonempirical ab initio calculations on structures, energies, and nonadditivity of the interaction. J Phy Chem B. 1997;101:9670–9677.

[40] Chen PP, Meng YM, Liu TYH, Peng W, Gao Y, He YQ, Qu RL, Zhang CY, Hu W, Ying BW. Sensitive urine immunoassay for visualization of lipoarabinomannan for noninvasive tuberculosis diagnosis. ACS Nano. 2023;17(7):6998–7006. 37010068 10.1021/acsnano.3c01374

