# Supplementary material for: Self-Assembled DNA Machine and Selective Complexation Recognition Enable Rapid Homogeneous Portable Quantification of Lung Cancer CTCs
Source: Research (Wash D C). 2024 Apr 18;7:0352. doi: 10.34133/research.0352 (PMC11070850; doi:10.34133/research.0352)
Supplement: Supplementary 1 — Figs. S1 to S4 Tables S1 to S5 [file research.0352.f1.docx]

**Supplementary Material**

**Self-Assembled DNA Machine and Selective Complexation Recognition Enable Rapid Homogeneous Portable Quantification of Lung Cancer CTCs**

Yue Wang^1^, Congcong Shen^1^, Chengyong Wu^1^, Zixuan Zhan^1^, Runlian Qu^1^, Yi Xie^1^, and Piaopiao Chen^1^*

^1^ Department of Laboratory Medicine, Med+X Center for Manufacturing, Department of Respiratory and Critical Care Medicine, Targeted Tracer Research and Development Laboratory, National Clinical Research Center for Geriatrics, West China Hospital, Sichuan University, Chengdu, Sichuan, 610041, China

**Corresponding author.* *E-mail: chenpp0828@wchscu.cn (P. Chen).*

**Content**

Title…………….……………….…………………………………………… ……..S-1

Content…………….……………….………………………………………..…….. .S-2

Materials and reagents…………….……………….………………………… ….....S-3

Table S1. Sequences of oligonucleotides used in the study…………………… ......S-4

Fig. S1. The chemical structure of calcein disodium salt. …………………….........S-5

Instruments………….…………….………………………………………………. .S-5

Scheme S1. Test strip preparation process and test strip size. ...................................S-6

Cell recovery, culture and passage….…………………………..…………………. .S-6

Analysis of mucin 1….…………….………………………...……………………. .S-7

Pretreatment steps of clinical samples. ………………………...…………………. .S-8

Agarose gel electrophoresis analysis. .......................................................................S-9

Optimization of mucin 1 analysis conditions. ….....….……………….…...…..….S-10

Fig. S2. Optimization of mucin 1 analysis conditions. ............................................S-11

Fig. S3. Analytical and selective performance of mucin 1.......................................S-11

Table S2. Comparison of different methods for the determination of mucin 1…....S-12

Table S3. Comparison of methods for the determination of CTCs…….….....…....S-12

Table S4. Diagnosis information and CTCs test results of clinical patients……….S-14

Fig. S4. The reproducibility and stability of handheld instrument...........................S-15

Table S5. FR-PCR kits and CTCs test results of clinical patients.………...………S-15

References……………………………………………………….…………………S-15

**Materials and Reagents.**

All oligonucleotides with different sequences were synthesized and purified by Sangon Biotechnology Co.; Ltd (Shanghai, China). The sequences of the oligonucleotides used in this work were shown in Table S1. Copper sulfate pentahydrate (CuSO_4_•5H_2_O) was ordered from Aladdin Reagent Co. (Shanghai, China). Sodium nitrate (NaNO_3_), HCl, NaOH, Mg(NO_3_)_2_, and nitric acid (HNO_3_) were purchased from Kelong Chemical Reagents (Chengdu, China). Transferrin, mucin 1, papain, thrombin, pepsin, trypsin, streptavidin (SA), glucose oxidase (GOD), human serum albumin (HSA), interferon *γ* (IFN-*γ*), HIV-1 p24, sodium pyrophosphate (PPi), and histidine (His) were ordered from Sigma-Aldrich (St. Louis, MO, USA).

Mucin 4 was brought from Shanghai Yu Bo Biotech Co., Ltd (Shanghai, China). Mucin 15, Mucin 16, Mucin 18 were supplied by Sangon Biotechnology Co.; Ltd (Shanghai, China). 3-(*N*-morpholino) propanesulfonic acid (MOPS) was purchased from Solarbio Technology Co.; Ltd (Beijing, China). All working solutions were prepared with a MOPS buffer solution (10 mM, 100 mM NaNO_3_, pH 7.6). Lymphatic separation fluid was purchased from Tianjin Haoyang Biological Manufacture Co., Ltd (Tianjin, China). Erythrocyte lysate was purchased from Solarbio Technology Co.; Ltd (Beijing, China). Phosphate buffered saline (PBS) was ordered from Corning (New York, USA). Fetal Bovine Serum (FBS) was ordered from ExCell Bio. (Shanghai, China). All reagents used in this work were of analytical or higher grade and used without further purification. Water used in all experiments was purified by a water purification system (Chengdu Ultrapure Technology Co.; Ltd.; Chengdu, China) with a resistivity of 18.25 MΩ·cm. Health human serum and whole blood of lung cancer patient samples were donated from the West China Hospital of Sichuan University (Chengdu, China, approval number: 20191045). Human Pulmonary Carcinoma Cell (A549), human hepatocellular carcinomas (HepG2), human normal mammary epithelial cells (MCF-10A), human umbilical vein endothelial cells (HUVEC), human embryonic kidney 293T (HEK-293T) cells and human normal lung epithelial cells (BEAS-2B) were provided by the Core Facilities of West China Hospital. All solutions were stored at 4 °C in a refrigerator until use.

**Table S1.** Sequences of oligonucleotides used in the study

| Names | Sequences (5'-3') |
| --- | --- |
| Aptamer | GCA GTT GAT CCT TTG GAT ACC CTG G |
| Y1 | AGT ACG CGT ACT CCA GGG TAT CCA AAG GAT CAA CTG C |
| Y2 | AGC TCG AGC TCA CGG CGA ATC TGA ACG ATA CCC TGG |
| Y3 | AGC TCG AGC TGC AGT TGA TCG ATT CGC CGT G |
| P1 | ACG GGC CAC ATC AAC TCA TTG ATA GAC AAT GCG TCC ACT GCC CGT |
| H1 | ATC AAT GAG TTG ATG TGG CCC GTC TAA GAA CGG GCC ACA TCA ACT |
| H2 | ACG GGC CAC ATC AAC TCA TTG ATA GTT GAT GTG GCC CGT TCT TAG |
| H3 | ACG GGC AGT GGA CGC ATT GTC TTC ATA CAG ACA ATG CGT CCA CT |
| H4 | AGA CAA TGC GTC CAC TGC CCG TAG TGG ACG CAT TGT CTG TAT GA |
| P2 | AGC AAG CAT TCA TAT TGG TTG GTG GAA GTG GGG GGG GTG GTG TA |
| H5 | Biotin-TTT TTT TTT TTT TTT TAC CAA TAT GAA TGC TTG CTC TCC GTA GCA AGC A TT CAT |
| H6 | Biotin-TTT TTT TTT TTT TTT TAG CAA GCA TTC ATA TTG GTA TGA ATG CTT GCT A CG GAG |
| H7 | Biotin-TTT TTT TTT TTT TTT TGG GGG GTG GTG TAC TAA GTT ACA CCA CCC CCC C CA CTT |
| H8 | Biotin-TTT TTT TTT TTT TTT TAC TTA GTA CAC CAC CCC CCA AGT GGG GGG GGT G GT GTA |


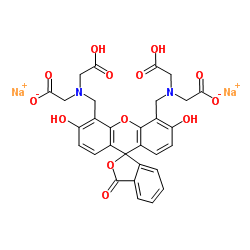


**Fig. S1**. The chemical structure of [calcein disodium salt](https://www.chemsrc.com/en/cas/108750-13-6_955452.html).

**Instruments.** The absorption and fluorescence spectrum of calcein was recorded by the Duetta Spectrophotometer (HORIBA Canada Inc). Chromatography paper (Whatman 3030-861, U.K.) was printed by inkjet printer (Hewlett Packard, hp1112) to serve as test strips. Cutting machine (Deli, Ningbo, China) was used to cut the chromatography paper. The MOPS buffer pH was determined using a PHS-3C Benchtop Ph meter (INESA, Shanghai, China). The scanning electron microscopy (SEM) images were carried on ZEISS Gemini 300 SEM (Germany) with the OXFORD Xplore. The transmission electron microscopy (TEM) images were obtained using a Tecnai G2F20 STWIN TEM at an accelerating voltage of 200 kV (FEI Co.; USA). The atomic force microscope (AFM) images were performed on Bruker Dimension Icon AFM (Germany). Dynamic Light Scattering (DLS) and Zeta potential assay was performed on Zeta potential analyzer (Nano ZSP, Malvern, England).

**Synthesis of CdTe QDs**. The CdTe QDs were synthesized referring to the previously reported method [1, 2]. First, a 50 mL solution contained CdCl_2_ (0.5 mmol) and trisodium citrate (0.2 g) was prepared. Then, MPA (52 μL) was instantly added into above solution, and the solution pH was adjusted to 10.5 with NaOH. Later, Na_2_TeO_3_ (0.1 mmol) and KBH_4_ (50 mg) were added into the above solution and refluxed for different time to obtain the CdTe QDs. Subsequently, high purity of CdTe QDs was obtained *via* precipitating with *n*-propanol and centrifuging (11000 rpm). The purified red CdTe QDs were redispersed in high-purity water before use.





**Scheme S1.** Test strip preparation process and test strip size.

**Cell Recovery.** The A549 cells were taken out of the liquid nitrogen tank, and shake quickly at 37 °C for 1-2 min, and then transferred to a centrifuge tube. 5 mL of dulbecco's modified eagle medium F-12 (DMEM/F-12) medium containing 10% fetal bovine serum was added and centrifuged at 1000 rpm for 3 min. The supernatant was discarded and 5 mL of medium was added to re-suspend by pipetting, followed by transfer to a petri dish. The cells were cultured at 37 °C, 5% CO_2_.

**Cell Culture and Passage.** Cell culture: The A549 cells were cultured in DMEM/F-12 medium containing 10% (v/v) FBS and 1% (v/v) penicillin-streptomycin solution and incubated at 37 °C with 5% CO_2_. When the cells were in the logarithmic growth phase, they were digested into a single cell suspension and counted. Finally, the cells were seeded in a petri dish at a density of 3000-5000 cells/well.

Cell passage: When the cells were in the logarithmic growth phase, the old medium was removed and washed with PBS buffer. Subsequently, it was digested with trypsin and centrifuged at 1000 rpm for 5 min to collect the cells. Then fresh medium was added and the cells were dispersed by pipetting. Finally, the cells were diluted with a ratio of 1:4 and placed in a new petri dish for continued culture.

**Analysis of Mucin 1.** The analysis of mucin 1 comprised the subsequent steps. First, the preparation of DNA nanospheres was carried out by mixing 30 μL of 50 μM Y1, Y2, and Y3 DNA strands in 210 μL of 10 mM 3-(*N*-morpholino) propanesulfonic acid (MOPS) buffer (10 mM, 100 mM NaNO_3_, pH 7.6). This mixture was heated to 95°C for 5 min and then gradually cooled to room temperature over a period of 3 h, forming the DNA nanosphere.

Following this, 10 μL of 12 μM aptamer-DNA, 40 μL of mucin 1 at various concentrations, and 40 μL of DNA nanospheres were added to 70 μL of 10 mM MOPS solution. This mixture was left to react at room temperature for 60 min. Subsequently, 10 μL of 5 μM CuSO_4_ solution was introduced to the reaction solution, which was left to react at room temperature for 50 min. Finally, 6 μL of 10 μM calcein disodium salt was added, and the fluorescence value was measured after 2 min of reaction.

**Pre-treatment Steps of Clinical Samples.** The clinical samples from peripheral blood were processed according to the following pre-treatment steps. First, 4 mL of whole blood was collected using a disposable airtight EDTA (ethylene diamine tetraacetic acid) anticoagulant vacuum blood collection tube (approved by West China Hospital of Sichuan University, Chengdu, China, approval number: 20191045). Then, 4 mL of phosphate-buffered saline (PBS) was added to 4 mL of whole blood, and the mixture was thoroughly mixed. Using two 15 mL centrifuge tubes, 4 mL of the blood and PBS mixture was added to each tube, followed by the gradual addition of 4 mL of lymphatic separation fluid. The resulting solution was then centrifuged at 4 °C for 20 min at 800 g, with an ascending speed of 1 and a descending speed of 1. As a result, the liquid in the centrifuge tube was separated into four layers from top to bottom: the first layer was plasma, the second layer was round milky lymphocytes, the third layer was transparent isolate, and the last layer consisted of red blood cells at the bottom. The second and third layers of liquid were collected and mixed thoroughly with equal volumes of PBS. A subsequent centrifugation at 4 °C was conducted at 2000 rpm for 7 min, with an ascending speed of 9 and a descending speed of 9. After discarding the supernatant, the precipitated cells were retained, and 5-8 mL of erythrocyte lysate was added. This mixture was then mixed and left to stand for 10 min. The above solution was once again centrifuged at 4 °C, this time at 500 g for 10 min, with an ascending speed of 9 and a descending speed of 9. Following the removal of the supernatant, the precipitate consisted of lymphocytes and free tumor cells, serving as the desired material for further analysis. Finally, 1 mL of fetal bovine serum (FBS) was added to obtain the test solution.

**Agarose Gel Electrophoresis Analysis.**

9 μL 4SGeIRed nucleic acid dye was added to form 2.5% agarose gel electrophoresis. In electrophoretic assay, each sample channel ended up containing 15 μL sample and 7 μL 6 x Loading buffer. Electrophoresis was performed in a 1 x TAE buffer and energized at 180 V for 50 min (Fig. 2P).

(1) Marker (25-500 bp)

(2) Aptamer (5 μΜ)

(3) Y1 (5 μΜ)

(4) Y2 (5 μΜ)

(5) Y3 (5 μΜ)

(6) DNA nanosphere (5 μΜ)

(7) DNA nanosphere + Aptamer (1 μΜ)

(8) DNA nanosphere + Aptamer (10 μΜ)

(9) DNA nanosphere + Aptamer (50 μΜ)

(10) DNA nanosphere + Aptamer (10 μΜ) + Mucin 1 (1 ag/mL)

(11) DNA nanosphere + Aptamer (10 μΜ) + Mucin 1 (100 ag/mL)

(12) DNA nanosphere + Aptamer (10 μΜ) + Mucin 1 (10 fg/mL)

9 μL 4SGeIRed nucleic acid dye was added to form 2.5% agarose gel electrophoresis. In electrophoretic assay, each sample channel ended up containing 16 μL sample and 4 μL 6 x Loading buffer. Electrophoresis was performed in a 1 x TAE buffer and energized at 180 V for 50 min (Fig. 2Q).

(1) Marker (25-500 bp)

(2) P2 (1 μΜ)

(3) H5 (1 μΜ)

(4) H6 (1 μΜ)

(5) H7 (1 μΜ)

(6) H8 (1 μΜ)

(7) H5 + H6 + H7 + H8 (1 μΜ)

(8) H5 + H6 + H7 + H8 + SA (1 μΜ)

(9) P2 (2 μΜ) + H5 + H6 + H7 + H8 (1 μΜ)

(10) P2 (2 μΜ) + H5 + H6 + H7 + H8 + SA (1 μΜ)

**Optimization of Mucin 1 Analysis Conditions.** The conditions for the analysis of mucin 1 were optimized. The maximum signal difference (blank *vs.* 10 fg/mL mucin 1) was achieved with a concentration of 12 μM of aptamer (Fig. S2A). A volume of 40 μL was selected as the amount of DNA nanospheres (5 μM), as this yielded a substantial signal difference (blank *vs.* 10 fg/mL mucin 1) (Fig. S2B and C). An optimal signal difference (blank *vs.* 10 fg/mL mucin 1) was attained when the reaction time between DNA nanospheres, mucin 1, and aptamer was set at 60 minutes (Fig. S2D). The concentration of Cu^2+^ was determined to be 5 μM (Fig. S2E and F), with a reaction time of 50 minutes for the interaction between Cu^2+^ and DNA (Fig. S2G and H). A volume of 6 μL of calcein (10 μM) was selected, and the reaction time was set at 2 minutes (Fig. S2I and J), resulting in maximum signal difference (blank *vs.* 10 fg/mL mucin 1) (Fig. S2K and L).





**Fig. S2**. Optimization of mucin 1 analysis conditions. (A) Amount of aptamer. (B) and (C) Amount of DNA nanosphere. (D) Incubation time of DNA nanosphere with aptamer and protein. (E) and (F) Amount of Cu^2+^. (G) and (H) Reaction time after adding Cu^2+^. (I) and (J) amount of calcein. (K) and (L) Reaction time of calcein. The error line represents the standard deviation of three repeated measurements.


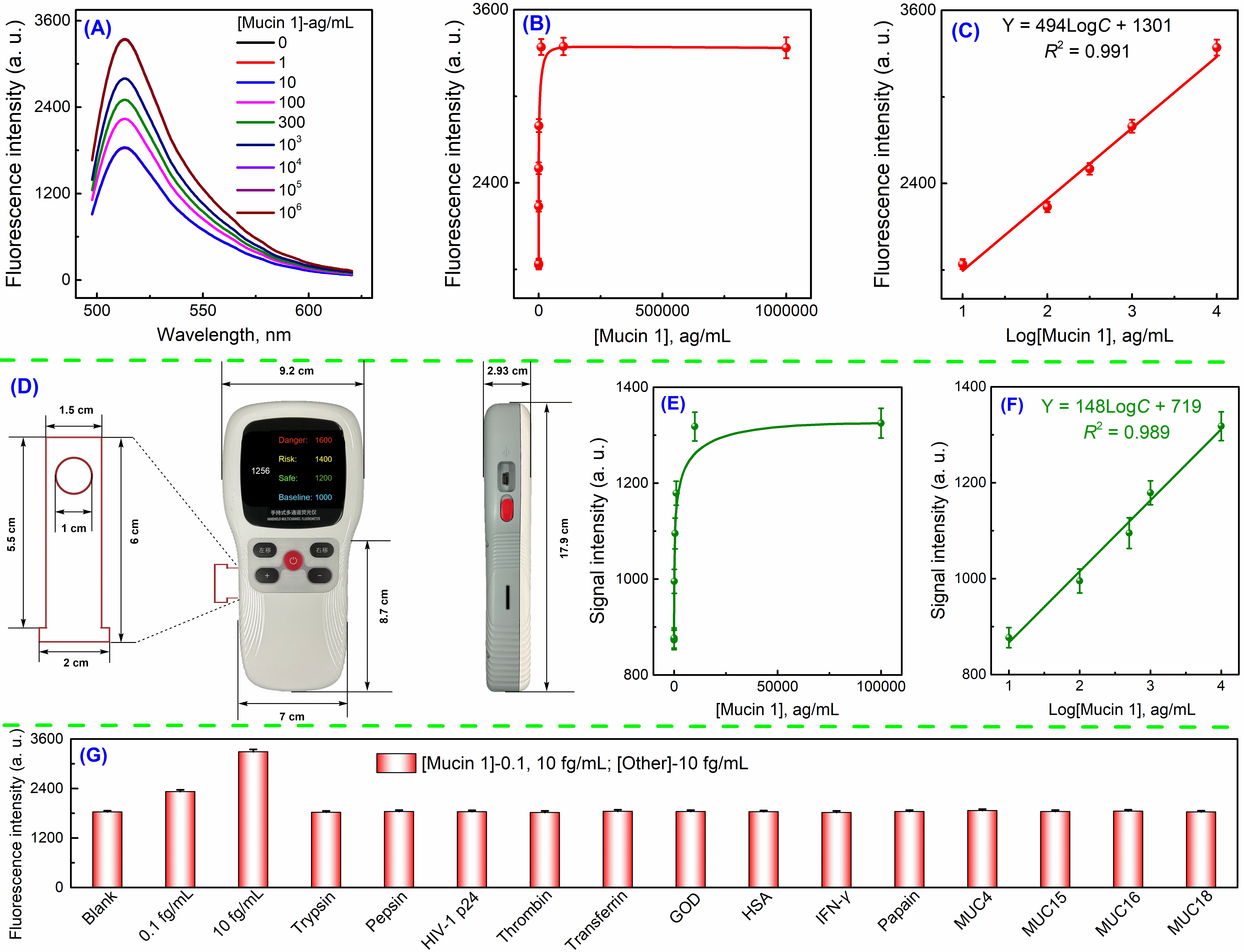


**Fig. S3.** Analytical and selective performance of mucin 1. (A) Fluorescence spectrum of calcein with different concentrations of mucin 1. (B, C) Fluorescence values and linear fitting of mucin 1 by fluorometer. (D) Dimensions of handheld fluorometer and test strips. (E, F) Fluorescence values and linear fitting of mucin 1 by handheld fluorometer. (G) Selectivity of mucin 1 homologues and other proteins. The error bars were calculated from three repetitions of the experiment.

**Table S2.** Comparison of different methods for the determination of mucin 1

| Method | System | Linear range; LOD | Reference |
| --- | --- | --- | --- |
| FRET^a^ | Aptamer; Au NBPs@PDA; Au NCs | 0.5-25 nM; 0.17 nM | [3] |
| Fluorescence | Aptamer; QDs; C-Ag^+^-C; CHA | 1 fg/mL-1 pg/mL; 0.15 fg/mL | [4] |
| Fluorescence | Aptamer; PDA; Cy3 | 1-50 nM; 0.8 nM | [5] |
| Fluorescence | MB; DNA G-quadruplex; Exo III; NMM | 50-1200 nM; 3.68 nM | [6] |
| EC^b^ | Immunoassay; PEI-AuNPs; SPCE | 0.1-100 ng/mL; 0.53 ng/mL | [7] |
| EC | Au-RGO; Fc-Aptamer; MB-Apt@Au | 1 pM-1 µM; 0.25 pM | [8] |
| ECL^c^ | Hf-TCBPE; Fc-HP_3_; Exo III | 1 fg/mL-1 ng/mL; 0.49 fg/mL | [9] |
| ECL | Aptamer; TbPO_4_: Ce | 1 fg/ml-10 ng /mL; 0.5 fg/mL | [10] |
| ECL | Porous carbon nanospheres; C-Au-Lum NSs; | 0.1 pg/mL-1 ng/mL; 47.6 fg/mL | [11] |
| ECL | Aptamer; V_2_O_5_ nanospheres; H_2_O_2_; CHA | 10 fg/mL-10 ng/mL; 3.33 fg/mL | [12] |
| SERS^d^ | Aptamer; Au nanostar; CHA | 1 fg/mL-0.01 ng/mL; 0.15 fg/mL | [13] |
| PEC^e^ | Aptamer; ZrO_2_ HSs; G-quadruplex | 1 fg/mL-10 ng/mL; 0.48 fg/mL | [14] |
| PEC | WO_3_/Fe_2_O_3_; AuNPs; HRP-SA; TMB | 10 fg/mL-100 ng/mL; 3.4 fg/mL | [15] |
| ICP-MS^f^ | Aptamer; QDs; C-Ag^+^-C | 2.5-15 ng/mL; 0.3 ng/mL | [16] |
| Nanopore | Aptamer; CuS NPs; Ag NPs; DNAzyme | 0.0005-0.5 pg/mL; 0.1 fg/mL | [17] |
| Fluorescence | Calcein; DNA nanosphere; aptamer; Cu^2+^ | 10 ag/mL-10 fg/mL; 3 ag/mL | This work |

^a^ Fluorescence resonance energy transfer (FRET); ^b^ Electrochemical; ^c^ Electrochemiluminescence; ^d^ surface-enhanced Raman scattering; ^e^ Photoelectrochemical; ^f^ Inductively coupled plasma mass spectrometry.

**Table S3.** Comparison of methods for the determination of CTCs (cells/mL)

| Method | System | Linear range; LOD | Verify practicality | Reference |
| --- | --- | --- | --- | --- |
| ICC^a^ (MCF-7) | NaEuF_4_ NPs; anti-EpCAM antibody | 2-1024; 10 | Blood samples of breast cancer patients | [18] |
| ICC (MGC-803  and BGC-823) | Aptamer; immunomagnetic particles | 5-500; 5 | Blood samples of gastric cancer patients | [19] |
| Fluorescence (A549) | Aptamer; CHA; QDs | 10-10^5^; 3 | Blood samples of lung cancer patients | [4] |
| Fluorescence (SMMC-7721) | Folate; terminal protection; TdT; Cu NPs; QDs | 1-100; 0.25 | Blood samples of liver cancer patients | [20] |
| Colorimetric (MCF-7) | Aptamer; SMCNTs; TMB | 10-500; 3 | Blood samples of breast, liver, lung, stomach, and colon cancers | [21] |
| Temperature (HeLa) | FA-PtCPs NRs; TMB | 10-10^5^; 2 | Human blood samples-spiked | [22] |
| EC^b^ (A549) | Aptamer; anti-EpCAM antibody; Branched PtAuRh trimetallic Nanospheres | 10-800; 1 | Blood samples of lung cancer patients | [23] |
| EC (CCRF-CEM) | Dendrimer-Au NPs network, aptamer | 300-1000; 80 | Human blood samples-spiked | [24] |
| EC (MCF-7) | Branched zwitterionic peptide; PANI films; aptamer | 50-10^6^; 20 | Human serum recovery | [25] |
| ECL^c^ (MCF-7) | Aptamer and anti-EpCAM antibody; Fe_3_O_4_@SiO_2_/Au/WO_3-x_ dots | 10-10^5^; 3 | Human blood samples-spiked | [26] |
| ECL (MCF-7) | Aptamer; Au@CDs; | 100-10^5^; 34 | Human serum recovery | [27] |
| SERS^d^ (MCF-7) | Aptamer; MB; RCA | 5-1000; 2 | Human blood samples-spiked | [28] |
| SERS (MCF-7) | Folate receptor; B-TiO_2_ NPs | 2-100; 2 | Blood samples of breast cancer patients | [29] |
| ICP-MS^e^ (MCF-7) | Aptamer; sea urchin-DMA-AuNPs; PTK7; EpCAM | Not mentioned; 0.1 | Blood samples of breast cancer patients | [30] |
| Nanopore (MCF-7) | Aptamer; DNA hydrogel; Ag NPs; CuS NPs | 5-2000; 2 | Blood samples of breast cancer patients | [17] |
| Nanopore (MCF-7) | Magnetic nanosphere; fluorescent nanosphere | 10-300; 5 | Blood samples of cancer patients | [31] |
| Imaging (BxPC-3, PANC-1 and Capan-1) | Aptamer; Fe_3_O_4_@DOP NPs; FAM; HCR | 100-10^5^; 21/41 | Staining of nude mouse tissue | [32] |
| Microfluidic chips | AP-Octopus-Chip; aptamer; AuNPs; GSH | 2-20; 2 | Blood samples of cancer patients | [33] |
| Fluorescence (A549) | Calcein; DNA nanosphere; aptamer; Cu^2+^ | 1-1000; 2 | Whole blood of lung cancer patients | This work |

^a^ Immunocytochemistry; ^b^ Electrochemical; ^c^ Electrochemiluminescence; ^d^ surface enhanced Raman scattering; ^e^ Inductively coupled plasma mass spectrometry.

**Table S4.** Diagnosis information and CTCs test results of clinical patients (cells/mL)

| No. | Age | Sex | Clinical diagnosis | FL | Handheld fluorometer | Consistency |
| --- | --- | --- | --- | --- | --- | --- |
| 1 | 51 | female | negative | <1 | <1 | yes |
| 2 | 58 | male | negative | <1 | <1 | yes |
| 3 | 45 | female | negative | <1 | <1 | yes |
| 4 | 54 | female | negative | <1 | <1 | yes |
| 5 | 57 | female | negative | <1 | <1 | yes |
| 6 | 48 | female | negative | <1 | NT^a^ | yes |
| 7 | 50 | female | negative | <1 | NT | yes |
| 8 | 27 | female | negative | <1 | NT | yes |
| 9 | 44 | female | negative | <1 | NT | yes |
| 10 | 65 | male | positive | <1 | NT | no |
| 11 | 66 | male | positive | 25 | NT | yes |
| 12 | 45 | female | positive | 4 | NT | yes |
| 13 | 54 | male | positive | 2 | 13 | yes |
| 14 | 69 | female | positive | 10 | 7 | yes |
| 15 | 23 | female | positive | 2 | 16 | yes |
| 16 | 60 | male | positive | 3 | 6 | yes |
| 17 | 54 | female | positive | 2 | 4 | yes |
| 18 | 40 | female | positive | 5 | 2 | yes |
| 19 | 26 | female | positive | 9 | 2 | yes |
| 20 | 30 | female | positive | 2 | 5 | yes |
| 21 | 35 | female | positive | <1 | 14 | no |
| 22 | 33 | male | positive | 6 | 8 | yes |
| 23 | 25 | female | positive | 2 | 3 | yes |
| 24 | 41 | female | positive | 11 | 3 | yes |
| 25 | 34 | female | positive | 3 | 2 | yes |
| 26 | 34 | female | positive | 99 | 5 | no |
| 27 | 63 | female | positive | 2 | 7 | yes |
| 28 | 65 | male | positive | <1 | NT | no |
| 29 | 55 | female | positive | 3 | NT | yes |
| 30 | 57 | female | positive | <1 | NT | no |
| 31 | 76 | male | positive | 3 | NT | yes |
| 32 | 49 | female | positive | 3 | NT | yes |
| 33 | 59 | male | positive | 2 | NT | yes |
| 34 | 71 | female | positive | 3 | NT | yes |
| 35 | 33 | female | positive | 3 | NT | yes |
| 36 | 73 | female | positive | 4 | NT | yes |
| 37 | 67 | male | positive | 5 | NT | yes |
| 38 | 59 | male | positive | 2 | NT | yes |
| 39 | 53 | female | positive | 2 | NT | yes |
| 40 | 67 | female | positive | 55 | NT | yes |
| 41 | 62 | female | positive | 3 | NT | yes |
| 42 | 56 | male | positive | 2 | NT | yes |
| 43 | 51 | female | positive | 8 | NT | yes |
| 44 | 27 | female | positive | 13 | NT | yes |
| 45 | 21 | female | positive | 18 | NT | yes |
| 46 | 43 | female | positive | 2 | NT | yes |

^a^ not test.





**Fig. S4.** The reproducibility and stability of handheld instrument. (A) The reproducibility of the handheld instrument was obtained by testing 10 samples of calcein. (B) Stability of the instrument was determined by testing calcein after different number of days. The error bars were derived from three repeated measurements.

**Table S5.** FR-PCR kits and CTCs test results of clinical patients

| No. | Age | Sex | FR^a^  (FU/3 mL) | Clinical diagnosis | CTCs  (this work, cells/mL) | Consistency |
| --- | --- | --- | --- | --- | --- | --- |
| 1 | 55 | female | 3.5 | negative | <1 | yes |
| 2 | 51 | male | 4.7 | negative | <1 | yes |
| 3 | 42 | male | 9.9 | positive | 4 | yes |
| 4 | 53 | female | 21.7 | positive | 17 | yes |
| 5 | 65 | male | 13.2 | positive | 10 | yes |

^a^ Folate receptor.

**References**

1. Chen PP, Meng YM, Liu TYH, Peng W, Gao Y, He YQ, Qu RL, Zhang CY, Hu W, Ying BW, Sensitive Urine Immunoassay for Visualization of Lipoarabinomannan for Noninvasive Tuberculosis Diagnosis. ACS Nano 2023;17:6998-7006.

2. Chen PP, Bai YJ, Tang SY, Wang N, He YQ, Huang K, Huang J, Ying BW, Cao Y, Homogeneous Binary Visual and Fluorescence Detection of Tetanus Toxoid in Clinical Samples Based on Enzyme-Free Parallel Hybrid Chain Reaction. Nano Lett. 2022;22:1710-1717.

3. Wang J, Gao YH, Liu PP, Xu SH, Luo XL, Core-Shell Multifunctional Nanomaterial-Based All-in-One Nanoplatform for Simultaneous Multilayer Imaging of Dual Types of Tumor Biomarkers and Photothermal Therapy. Anal. Chem. 2020;92:15169-15178.

4. Chen PP, Wang Y, He YQ, Huang K, Wang X, Zhou RH, Liu TYH, Qu RL, Zhou J, Peng W, Li M, Bai YJ, Chen J, Huang J, Geng J, Xie Y, Hu W, Ying BW, Homogeneous Visual and Fluorescence Detection of Circulating Tumor Cells in Clinical Samples *via* Selective Recognition Reaction and Enzyme-Free Amplification. ACS Nano 2021;15:11634-11643.

5. Wang D-E, Gao XH, You SQ, Chen M, Ren L, Sun WJ, Yang H, Xu HY, Aptamer-Functionalized Polydiacetylene Liposomes Act as a Fluorescent Sensor for Sensitive Detection of MUC1 and Targeted Imaging of Cancer Cells. Sens. Actuators, B 2020;309:127778.

6. Shi H, Jin T, Zhang JW, Huang XT, Tan CY, Jiang YY, Tan Y, A Novel Aptasensor Strategy for Protein Detection based on G-Quadruplex and Exonuclease III-Aided Recycling Amplification. Chinese Chem. Lett. 2020;31:155-158.

7. Kuntamung K, Jakmunee J, Ounnunkad K, A Label-Free Multiplex Electrochemical Biosensor for the Detection of Three Breast Cancer Biomarker Proteins Employing Dye/Metal Ion-Loaded and Antibody-Conjugated Polyethyleneimine-Gold Nanoparticles. J. Mater. Chem. B 2021;9:6576-6585.

8. Yang SH, Zhang FF, Liang QL, Wang ZH, A Three-Dimensional Graphene-Based Ratiometric Signal Amplification Aptasensor for MUC1 Detection. Biosens. Bioelectron. 2018;120:85-92.

9. Huang W, Hu G-B, Yao L-Y, Yang Y, Liang W-B, Yuan R, Xiao D-R, Matrix Coordination-Induced Electrochemiluminescence Enhancement of Tetraphenylethylene-Based Hafnium Metal-Organic Framework: An Electrochemiluminescence Chromophore for Ultrasensitive Electrochemiluminescence Sensor Construction. Anal. Chem. 2020;92:3380-3387.

10. Wang C, Han Q, Liu PK, Zhang G, Song L, Zou XC, Fu YZ, Novel Enhanced Lanthanide Electrochemiluminescence Luminophores: Ce^3+^-Doped TbPO_4_ Facile Synthesis and Detection for Mucin1. Anal. Chem. 2021;93:12289-12295.

11. Gao J-W, Chen M-M, Wen W, Zhang XH, Wang SF, Huang W-H, Au-Luminol-Decorated Porous Carbon Nanospheres for the Electrochemiluminescence Biosensing of MUC1. Nanoscale 2019;11:16860-16867.

12. Yang F, Jiang XY, Zhong X, Wei SP, Yuan R, Highly Sensitive Electrochemiluminescence Detection of Mucin1 based on V_2_O_5_ Nanospheres as Peroxidase Mimetics to Catalyze H_2_O_2_ for Signal Amplification. Sens. Actuators, B 2018;265:126-133.

13. Fan GL, Gao XR, Xu SL, Li X, Zhang Q, Dai CF, Xue QW, Wang HS, Engineering an Au Nanostar-Based Liquid Phase Interfacial Ratiometric SERS Platform with Programmable Entropy-Driven DNA Circuits to Detect Protein Biomarkers in Clinical Samples. Chem. Commun. 2022;58:407-410.

14. Fu YM, Yu Q, Zhang QQ, Zhang XH, Du CC, Chen JH, A Photocurrent-Polarity-Switching Biosensor for Highly Selective Assay of Mucin 1 based on Target-Induced Hemin Transfer from ZrO_2_ Hollow Spheres to G-Quadruplex Nanowires. Biosens. Bioelectron. 2021;192:113547.

15. Sun JL, Li L, Ge SG, Zhao PN, Zhu PH, Wang ML, Yu JH, Dual-Mode Aptasensor Assembled by a WO_3_/Fe_2_O_3_ Heterojunction for Paper-Based Colorimetric Prediction/Photoelectrochemical Multicomponent Analysis. ACS Appl. Mater. Interfaces 2021;13:3645-3652.

16. Wang X, Chen X, Zhou RH, Hu PY, Huang K, Chen PP, Filter-Assisted Separation of Multiple Nanomaterials: Mechanism and Application in Atomic/Mass Spectrometry/Fluorescence Label-Free Multimode Bioassays. Anal. Chem. 2021;93:3889-3897.

17. Sun K, Chen PP, Yan SX, Yuan WD, Wang Y, Li XQ, Dou LQ, Zhao CJ, Zhang JF, Wang Q, Fu ZK, Wei L, Xin ZD, Tang ZY, Yan YC, Peng YM, Ying BW, Chen J, Geng J, Ultrasensitive Nanopore Sensing of Mucin 1 and Circulating Tumor Cells in Whole Blood of Breast Cancer Patients by Analyte-Triggered Triplex-DNA Release. ACS Appl. Mater. Interfaces 2021;13:21030-21039.

18. Guo HH, Song XR, Lei W, He C, You WW, Lin QZ, Zhou SY, Chen XY, Chen Z, Direct Detection of Circulating Tumor Cells in Whole Blood Using Time-Resolved Luminescent Lanthanide Nanoprobes. Angew. Chem. Int. Ed. 2019;58:12195-12199.

19. Li CL, Yang SH, Li R, Gong SY, Huang M, Sun YQ, Xiong GX, Wu DP, Ji MJ, Chen Y, Gao C, Yu YY, Dual-Aptamer-Targeted Immunomagnetic Nanoparticles to Accurately Explore the Correlations between Circulating Tumor Cells and Gastric Cancer. ACS Appl. Mater. Interfaces 2022;14:7646-7658.

20. Chen PP, He YQ, Liu TYH, Li FL, Huang K, Tang D, Jiang PJ, Wang SJ, Zhou J, Huang J, Xie Y, Wei YG, Chen J, Hu W, Ying BW, Homogeneous Two-Dimensional Visual and Fluorescence Analysis of Circulating Tumor Cells in Clinical Samples *via* Steric Hindrance Regulated Enzymes Recognition Cleavage and Elongation. Biosens. Bioelectron. 2022;202:114009.

21. Zhu L, Feng XQ, Yang SH, Wang JY, Pan YX, Ding JH, Li CL, Yin XX, Yu YY, Colorimetric Detection of Immunomagnetically Captured Rare Number CTCs using MDNA-Wrapped Single-Walled Carbon Nanotubes. Biosens. Bioelectron. 2021;172:112780.

22. Li XY, Lu SY, Mu XJ, Li TR, Sun SH, Zhao Y, Hai J, Wang BD, Red-Light-Responsive Coordination Polymers Nanorods: New Strategy for Ultrasensitive Photothermal Detection of Targeted Cancer Cells. Biosens. Bioelectron. 2021;190:113417.

23. Cai J, Shen HW, Wang YH, Peng Y, Tang ST, Zhu YL, Liu Q, Li BY, Xie GM, Feng WL, A Dual Recognition Strategy for Accurate Detection of CTCs Based on Novel Branched PtAuRh Trimetallic Nanospheres. Biosens. Bioelectron. 2021;176:112893.

24. Wang C, Zhao X-P, Liu F-F, Chen YM, Xia X-H, Li J, Dendrimer-Au Nanoparticle Network Covered Alumina Membrane for Ion Rectification and Enhanced Bioanalysis. Nano Lett. 2020;20:1846-1854.

25. Liu NZ, Song JY, Lu YW, Davis JJ, Gao FX, Luo XL, Electrochemical Aptasensor for Ultralow Fouling Cancer Cell Quantification in Complex Biological Media Based on Designed Branched Peptides. Anal. Chem. 2019;91:8334-8340.

26. Pan D, Fang ZZ, Yang EL, Ning ZQ, Zhou Q, Chen KY, Zheng YJ, Zhang YJ, Shen YF, Facile Preparation of WO_3-x_ Dots with Remarkably Low Toxicity and Uncompromised Activity as Co-reactants for Clinical Diagnosis by Electrochemiluminescence. Angew. Chem. Int. Ed. 2020;59:16747-16754.

27. Liu PF, Wang L, Zhao KR, Liu ZJ, Cao HX, Ye SY, Liang GX, High Luminous Efficiency Au@CDs for Sensitive and Label-Free Electrochemiluminescent Detection of Circulating Tumor Cells in Serum. Sens. Actuators, B 2020;316:128131.

28. Li JX, Dong C, Gan HY, Gu XY, Zhang JJ, Zhu YF, Xiong JR, Song CY, Wang LH, Nondestructive Separation/Enrichment and Rolling Circle Amplification-Powered Sensitive SERS Enumeration of Circulating Tumor Cells *via* Aptamer Recognition. Biosens. Bioelectron. 2023;231:115273.

29. Xu XW, Lin J, Guo YH, Wu XX, Xu YP, Zhang DH, Zhang XZ, Yujiao X, Wang J, Yao CY, Yao JL, Xing J, Cao Y, Li YY, Ren WZ, Chen TX, Ren Y, Wu AG, TiO_2_-Based Surface-Enhanced Raman Scattering Bio-Probe for Efficient Circulating Tumor Cell Detection on Microfilter. Biosens. Bioelectron. 2022;210:114305.

30. Zhang X, Wei X, Men X, Wu C-X, Bai J-J, Li W-T, Yang T, Chen M-L, Wang J-H, Dual-Multivalent-Aptamer-Conjugated Nanoprobes for Superefficient Discerning of Single Circulating Tumor Cells in a Microfluidic Chip with Inductively Coupled Plasma Mass Spectrometry Detection. ACS Appl. Mater. Interfaces 2021;13:43668-43675.

31. Wu L-L, Wen C-Y, Hu J, Tang M, Qi C-B, Li N, Liu C, Chen L, Pang D-W, Zhang Z-L, Nanosphere-Based One-Step Strategy for Efficient and Nondestructive Detection of Circulating Tumor Cells. Biosens. Bioelectron. 2017;94:219-226.

32. Dong Q, Jia XN, Wang YL, Wang H, Liu Q, Li D, Wang J, Wang EK, Sensitive and Selective Detection of Mucin1 in Pancreatic Cancer using Hybridization Chain Reaction with the assistance of Fe_3_O_4_@Polydopamine Nanocomposites. J. Nanobiotechnol. 2022;20:94:

33. Song YL, Shi YZ, Huang MJ, Wang W, Wang Y, Cheng J, Lei ZC, Zhu Z, Yang CY, Bioinspired Engineering of a Multivalent Aptamer-Functionalized Nanointerface to Enhance the Capture and Release of Circulating Tumor Cells. Angew. Chem. Int. Ed. 2019;58:2236-2240.
